# Phylogenetic and Spatiotemporal Analyses of the Complete Genome Sequences of Avian Coronavirus Infectious Bronchitis Virus in China During 1985–2020: Revealing Coexistence of Multiple Transmission Chains and the Origin of LX4-Type Virus

**DOI:** 10.3389/fmicb.2022.693196

**Published:** 2022-04-04

**Authors:** Wensheng Fan, Jiming Chen, Yu Zhang, Qiaomu Deng, Lanping Wei, Changrun Zhao, Di Lv, Liting Lin, Bingsha Zhang, Tianchao Wei, Teng Huang, Ping Wei, Meilan Mo

**Affiliations:** College of Animal Science and Technology, Guangxi University, Nanning, China

**Keywords:** infectious bronchitis virus, recombination, selective pressure, Bayesian phylogenetics, viral phylodynamics, spatial transmission

## Abstract

Infectious bronchitis (IB) virus (IBV) causes considerable economic losses to poultry production. The data on transmission dynamics of IBV in China are limited. The complete genome sequences of 212 IBV isolates in China during 1985–2020 were analyzed as well as the characteristics of the phylogenetic tree, recombination events, dN/dS ratios, temporal dynamics, and phylogeographic relationships. The LX4 type (GI-19) was found to have the highest dN/dS ratios and has been the most dominant genotype since 1999, and the Taiwan-I type (GI-7) and New type (GVI-1) showed an increasing trend. A total of 59 recombinants were identified, multiple recombination events between the field and vaccine strains were found in 24 isolates, and the 4/91-type (GI-13) isolates were found to be more prone to being involved in the recombination. Bayesian phylogeographic analyses indicated that the Chinese IBVs originated from Liaoning province in the early 1900s. The LX4-type viruses were traced back to Liaoning province in the late 1950s and had multiple transmission routes in China and two major transmission routes in the world. Viral phylogeography identified three spread regions for IBVs (including LX4 type) in China: Northeastern China (Heilongjiang, Liaoning, and Jilin), north and central China (Beijing, Hebei, Shanxi, Shandong, and Jiangsu), and Southern China (Guangxi and Guangdong). Shandong has been the epidemiological center of IBVs (including LX4 type) in China. Overall, our study highlighted the reasons why the LX4-type viruses had become the dominant genotype and its origin and transmission routes, providing more targeted strategies for the prevention and control of IB in China.

## Introduction

Avian infectious bronchitis (IB) is an economically critical poultry disease worldwide. IB virus (IBV) is a member of the *Coronaviridae* family. Coronavirus infects humans and many other animals. Since the beginning of this century, three coronaviruses (SARS-CoV, MERS-CoV, and SARS-CoV-2) have infected humans and caused death (Drosten et al., 2003; Zaki et al., 2012; Lauer et al., 2020). In particular, the COVID-19 outbreak caused by SARS-CoV-2 in late 2019 has created a serious public health problem worldwide. Since people have close contact with animals, and coronavirus is prone to mutation and recombination, it is unpredictable whether recombination will occur between SARS-CoV-2 and animal coronavirus. Therefore, it is necessary to strengthen the prevention and control of animal coronavirus infections, including IB.

Infectious bronchitis virus has a single-stranded positive-sense RNA genome of approximately 27.6 kilobases (kb) in length encoding four structural proteins: spike (S) glycoprotein, envelope (E) protein, membrane (M) glycoprotein, and nucleocapsid (N) protein (Cavanagh, 2007; Fan et al., 2019). The S protein is cleaved into S1 and S2 subunits by furin-like host cell proteases (Cavanagh et al., 1992). The S1 protein mediates the binding of the virus to host cells and is the most variable region, which contains serotype-specific and virus-neutralizing epitopes (Ignjatovic and Sapats, 2005). Therefore, the sequence characteristics and evolution rules of S1 gene are often used for molecular epidemiological analysis of IBV.

Multiple IBV genotypes, serotypes, or variants have been identified globally. IBV strains worldwide are classified into seven genotypes comprising thirty-five distinct viral lineages based on the complete S1 gene sequences (Molenaar et al., 2020). In China, the IBV strains are divided into multiple distinct groups, and the LX4 type (QX or GI-19) appears to be the dominant genotype (Xu et al., 2018). The LX4-type strain was first isolated in China in 1996 (Wang et al., 1997) and has spread to several neighboring countries and even to Europe (Liu et al., 2006; Huang et al., 2019). At present, LX4-type viruses have already become the dominant epidemic strains in the world (Zhao et al., 2017; Huang et al., 2019). However, where did the LX4 type come from? Why did the LX4 type evolve into a major dominant epidemic strain in the world? These problems still remain unclear. Further investigation is needed.

As mentioned above, many molecular epidemiological studies based on S1 gene of IBV have been reported (Valastro et al., 2016; Bande et al., 2017; Huang et al., 2019; Molenaar et al., 2020). However, these studies could not fully explain the changes in the pathotypes and serotypes of IBV variants (Lin and Chen, 2017; Fan et al., 2019). Recently, there have been some studies based on the variation of other structural proteins of IBV (Finger et al., 2018; Yuan et al., 2018; Liang et al., 2019; Wu et al., 2019), indicating that these proteins also play important roles in the evolution of IBV. However, the abovementioned studies mainly focused on S1 gene, occasionally M and N genes, but rarely analyzed all the four structural protein genes and the full-length genome. In order to obtain comprehensive genetic information and molecular mechanism of variation of circulating isolates, it is necessary to analyze all the structural proteins of IBV simultaneously and even the whole genome.

Recombination is an important contributing factor in the emergence and evolution of IBV or even the emergence of new coronaviruses or novel diseases (Jackwood et al., 2010). Recombination events could occur between the field and vaccine viruses or between the field viruses (Fan et al., 2019). So far, the recombinants between the field and vaccine viruses were common. However, there are multiple live-attenuated vaccines used in the field in China, and little attention has been paid to which live vaccine is more likely to participate in the recombination. In addition, many previous recombination descriptions merely analyzed the recombination of the S1 or S genes, but the S1 or S genes were only a small part of the IBV genome and could not represent all the biological characteristics and recombination events of the whole genome, resulting in the failure to fully understand the evolution rules of IBV. Moreover, few studies on recombination hotspots are available. Hence, a thorough and comprehensive recombination analysis using the full genome sequences of as many strains as possible is needed.

China is the major producer of poultry worldwide with so many chicken breeds, differing ages, and a wide variety of rearing patterns (Huang et al., 2019). Yellow chickens are the major local breeds in southern China where about 5 billion birds were produced in 2019 (Deng et al., 2021). Compared to the white-feather chickens, the yellow chickens have a long raising time (about 120 days), and the free-range style is used with ineffective biosecurity measures due to the open fields and mountainsides (Wei, 2019; Deng et al., 2021). Moreover, some other inadequate biosecurity measures were applied in these flocks, such as lack of following the all-in, all-out basis, multi-age chicken farming, close distance between different-age flocks, different vaccination programs, and incomplete disinfection between each batch of the birds (Deng et al., 2021), which increase the odds of multiple infections of several IBV strains. In recent years, due to the frequent vaccination failure, many chicken farms tended to administer more frequent immunization and simultaneous administration of multiple-type live-attenuated vaccines, which promoted more frequent recombinations and mutations. Therefore, the prevention and control of IB are facing greater challenges and difficulties. In order to provide more targeted strategies to prevent and control IB, a whole-genome sequence analysis with longer time spans, that is more comprehensive, and with more isolates is needed.

Although there were many previous studies that focused on the genetic analysis of IBVs, most of the studies focused on the phylogenetic and recombination analyses of S1 gene (Yan et al., 2019; Ren et al., 2020; Zhang et al., 2020). The number of IBV strains and the spanning time were limited. There were few studies on the issues of defining the history of IBV and the spread trajectories in China. There was little information about its population dynamics over time and the major routes of its domestic spread at the national level. It is necessary to carry out long-term, in-depth, systematic epidemiological monitoring of IBVs in China. Hence, we conducted the analyses of phylogenetic, recombination, dN/dS ratios, temporal dynamics, phylogeographic analysis, and demographic history of 212 IBV strains isolated in China during the years 1985–2020 in the present study, aiming to obtain a comprehensive insight into the evolution and spread of IBV in China and provide more targeted strategies for the prevention and control of IB in China and even around the world, as well as a reference for the prevention and control of other animal coronavirus diseases and even the COVID-19 pandemic.

## Materials and Methods

### Infectious Bronchitis Virus Isolates and Sequence Data

All complete genome sequences of Chinese IBV isolates were downloaded from the GenBank database on December 31, 2020^1^. Only the complete genome sequences with known sampling information, including dates (years) and locations (provinces) were selected from the GenBank database. On this basis, those sequences with a length greater than 27,000 nt were further selected for analysis to ensure robust statistical support. The complete genome sequences of 212 IBV isolates from 1985 to 2020 were available for analysis after the abovementioned screening (Supplementary Tables 1, 2). According to the GenBank database, the whole-genome sequences of 212 Chinese IBV isolates were obtained by Sanger dideoxy sequencing technology. The earliest eligible IBV strain sequence available from the GenBank database was GX-C isolated in 1985, which was sequenced and submitted to the GenBank database by our group (Mo et al., 2013; Fan et al., 2019). The digital map (Supplementary Figure 1) was obtained from the Institute of Geographic Sciences and Natural Resources Research in China (resource and environment data cloud platform^2^). These 212 IBV isolates were isolated in 22 provinces or autonomous cities (zones) with a wide geographical range in China (Supplementary Figure 1). The available complete sequences of 33 reference IBV strains (Supplementary Table 3) included the commonly used live vaccine strains in China, and the dominant genotypes and serotypes around the world (Thor et al., 2011; Mo et al., 2013; Zhao et al., 2016, 2017; Fan et al., 2019; Huang et al., 2020). Among all complete genome sequences of IBV isolates from other countries retrieved from the GenBank database, 68 IBV isolates with known sampling dates and geographic locations were chosen for analysis together (Supplementary Tables 4, 5). Different datasets, including the sequences of the S1, S2, E, M, and N structural genes, were extracted from the complete genome sequences and used for analyses.

### Gene Alignment and Phylogenetic Analysis

The complete genome sequences and structural genes S1, S2, E, M, and N of 212 Chinese isolates and 33 reference strains were aligned using the Mafft version 7^3^ (Katoh and Standley, 2013). The lowest Bayesian information criterion (BIC) was calculated using Jmodeltest^4^ (Darriba et al., 2012). The best-fitting substitution model (GTR + G + I) was selected. Phylogenetic trees based on 212 Chinese isolates and 33 reference strains were constructed using MEGA version 6.06 according to the previous description (Tamura et al., 2013). Phylogenetic trees based on the complete genome sequences of 68 IBV isolates from other countries were also constructed as the above method.

Alignment of the deduced amino acid (aa) sequences of the S1 protein was performed for the 212 Chinese isolates by comparing with the vaccine strains M41, H120, 4/91, QXL87, and LDT3-A, which were commonly used in China, and a field strain LX4, representing the dominant IBV genotype circulating in China. The minimal receptor-binding domain (RBD) and three hypervariable regions (HVRs) were compared by the aa sequences of S1 protein.

### Recombination Detection

Two different approaches were used to identify the potential recombination events that happened among the IBV complete genomes, S1, S2, E, M, and N genes of the 212 Chinese isolates. First, the complete genomes and the five genes were detected for potential recombinants in the sequence alignment using seven methods (RDP, GENECONV, BOOTSCAN, MAXCHI, CHIMAERA, SISCAN, and 3SEQ) available in the Recombination Detection Program (RDP4, Version 4.95, Simmonics, University of Warwick, Coventry, United Kingdom) (Martin, 2009). To minimize false positives for the complete genomes, recombination events were only considered to be significant if they were supported by at least four of the seven methods with a significance value lower than 10^–12^ (*p*-value < 10^–12^) (Thor et al., 2011). To minimize false positives for the S1, S2, E, M, and N genes, recombination events were only considered to be significant if they were supported by at least four of the seven methods with a significance value lower than 10^–6^ (*p*-value < 10^–6^) (Franzo et al., 2017). Second, the complete genomes and the five genes further verified the potential recombination events and the breakpoints by similarity plots (SimPlots) analysis in SimPlot version 3.5.1 (Fan et al., 2019), with a window of 200 bp and step size of 20 bp. Both Simplot and RDP4 statistically identified each of the beginning and ending breakpoints (with no gaps) of the recombinant strains.

### Estimation of dN/dS Ratios

The dN/dS ratios within the proteins S1, S2, E, M, and N from the 212 Chinese IBV isolates were analyzed by the SLAC, FEL, and IFEL methods of Datamonkey version^5^ (Jackwood and Lee, 2017). The dN/dS ratios of the S1 protein of different genotypes of IBV were also analyzed as the abovementioned method. The maximum likelihood (ML) phylogenetic trees and the best-fit model (GTR + G + I) of nucleotide substitution were used for analyzing all the proteins. The recombinants were excluded in order to reduce false detection of the dN/dS ratios.

### Temporal Dynamics of Infectious Bronchitis Virus

To infer the evolutionary rate, the time to the most recent common ancestor (tMRCA), and population dynamics of the Chinese IBV, the Bayesian Markov chain Monte Carlo (MCMC) implemented in BEAST version 1.10.4 package^6^ (Suchard et al., 2018) was used. The recombinants were excluded in order to reduce the effect of evolutionary parameter estimation. To test the temporal signal in the dataset, a regression of root-to-tip genetic distances against the year of sampling in TempEst v 1.5.1^7^ (Rambaut et al., 2016) was performed using the unrooted ML tree generated with IQ-TREE v1.6.12 (Nguyen et al., 2015). The best-fitting root of the tree that optimizes the temporal signal was selected. The GTR + G + I substitution models were used for the complete genome sequences based on the result of jModelTest. BEAST version 1.10.4 package was set to both the strict molecular clock model and the uncorrected lognormal relaxed molecular clock model with two demographic models (constant size and Bayesian skyline). Four pairs of models were generated, and each pair was run for 5 million generations with every 50,000 cycles sampled to ensure the convergence of relevant parameters (i.e., effective sample sizes [ESSs] > 200) using Tracer v1.7 (Rambaut et al., 2018). The fitting clock rate and virus population evolutionary model were compared using ACIM, and a strict molecular clock and Bayesian skyline were selected (Supplementary Table 6). The MCMC was run for at least 500 million generations, allowing 10% burn-in and sub-sampling every 5 million steps. Convergence was evaluated by calculating the ESS of the parameters with ESS > 200 using Tracer v1.7 (Rambaut et al., 2018). The tree was summarized as maximum clade credibility (MCC) tree using Tree Annotator v1.10.4, and then the created MCC tree was visualized using FigTree v1.4.3. The change of effective population size over time was inferred by Bayesian skyline plots (Rambaut et al., 2018).

### Phylogeographic Analysis and Demographic History of Infectious Bronchitis Virus

To gain insight into the circulation of IBV across the poultry producing regions in China, the spatial transmission patterns were reconstructed using a phylogeographic analysis (Baele et al., 2012) in BEAST 1.10.4, based on the dataset of complete genome sequences of Chinese IBV isolates. To infer the diffusion rates among geographic locations, the asymmetric substitution model with the Bayesian stochastic search variable selection (BSSVS) was selected in BEAST v1.10.4, also allowing Bayes factors (BF) calculations to test for significant diffusion rates in SPREAD3 v0.9.7 (Bielejec et al., 2016). Spread pathways were considered to be important when they yielded a BF greater than 3 and when the mean posterior value of the corresponding was greater than 0.50. The MCMC simulations for 500 million steps across three independent Markov chains were run, and samples of every 50,000 steps were collected. The ESS of each parameter was also required to be more than 200.

In order to further explore the evolutionary origin of the LX4 type of IBV, phylogeographic analysis of the LX4-type viruses was performed based on the complete genome sequences of IBV from China and other countries. The MCMC analysis was run for 300 million steps with a sampling of every 30,000 steps. Other parameters were the same as the above method.

## Results

### Phylogenetic Analysis

**FIGURE 1:**
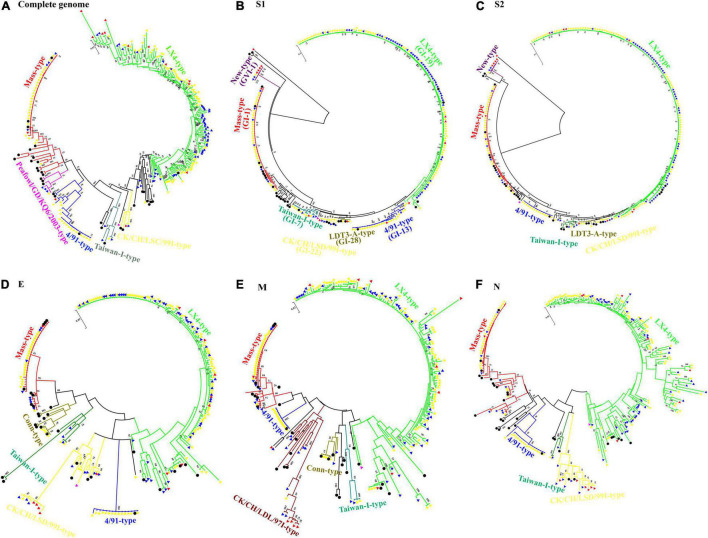
Phylogenetic trees of the complete genome **(A)** and genes S1 **(B)**, S2 **(C)**, E **(D)**, M **(E)**, and N **(F)** of infectious bronchitis viruses (IBVs). Each type of IBV was highlighted in different colors. The 33 IBV reference strains were marked with filled •. IBV isolates isolated during 1985–1998 were marked with filled 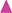. The 69 IBV isolates isolated during 1999–2010 were marked with filled 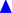. The 128 IBV isolates isolated during 2011–2014 were marked with filled 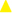, and the 14 IBV isolates isolated during 2015–2020 were marked with filled 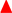. Phylogenetic trees were constructed with the maximum likelihood method using MEGA version 6.06. The bootstrap clade support values were computed based on 1,000 bootstrap replicates. The number represents the percentage of times that the clade appeared in the bootstrap tree distribution. Bootstrap values greater than 50% are shown.

Based on the phylogenetic tree analyses of the complete genome and S1, S2, E, M, and N genes, the 212 Chinese IBV isolates were segregated into 6, 7, 7, 6, 6, and 5 distinct groups, respectively (Figures 1A–F). There were 71.70% (152/212), 56.60% (120/212), 63.68% (135/212), 64.62% (137/212), 64.62% (137/212), and 66.04% (140/212) of the IBV isolates clustered in the LX4 type, respectively according to the complete genome and S1, S2, E, M, and N genes. The LX4 type has been the predominant genotype since 1999 (Supplementary Figure 2). Among the complete genome sequences of 68 IBV isolates from other countries, 15 isolates (22.06%) belonged to the LX4 type (Supplementary Figure 3). The phylogenetic tree based on the complete genome sequences showed that all the Chinese IBV isolates were segregated into the LX4 type, Mass type, 4/91 type, Peafowl/GD/KQ6/2003 type, Taiwan-I type, and CK/CH/LSC/99I type, except a unique isolate GX-C (Figure 1A). According to the phylogenetic tree characteristics of the complete genome sequence of 212 Chinese IBV isolates analyzed in this study, the LX4 type was the predominant genotype since 1999; the Mass type, 4/91 type, and Taiwan-I type were the second, third, and fourth most dominant genotypes, respectively, but the Mass type and 4/91 type nearly disappeared after 2015. The phylogenetic tree based on S1 gene sequences (Figure 1B) showed that all the Chinese isolates were segregated into the LX4 type (GI-19, 56.60%, 120/212), Mass type (GI-1, 18.87%, 40/212), 4/91 type (GI-13, 11.32%, 24/212), Taiwan-I type (GI-7, 5.19%, 11/212), New type (GVI-1, 3.77%, 8/212), CK/CH/LSC/99I type (GI-22, 2.36%, 5/212), and LDT3-A type (GI-28, 1.4%, 3/212), respectively, except for the unique isolate GX-C. The LX4 type was the predominant genotype since 1999. The Mass type and the 4/91 type were the second and third most dominant genotypes, and their change trends were similar to those based on the complete genome sequences. However, the Taiwan-I type (GI-7) has increased since 2011, and the New type (GVI-1) increased since 2015.

The alignment results of S1 gene sequences with those of the vaccine strain M41, H120, 4/91, QXL87, LDT3-A, and the reference field strain LX4 revealed the presence of multiple mutations (Supplementary Figure 4). There was a 5-aa-insertion (FFLII), located in the HVR I found in S1 genes of 11 isolates.

### Analysis of Recombinants

**FIGURE 2:**
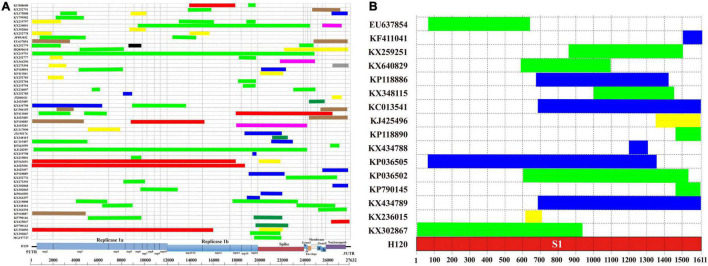
The vaccine strain H120 was used as the reference strain. **(A)** The virus genome of the coronavirus avian infectious bronchitis showing the locations of 5′ and 3′ UTRs and the coding regions. **(B)** Recombination regions identified across S1 gene. Recombinant regions detected by RDP4 are highlighted in the line. The green, yellow, blue, red, dark brown, pink, dark green, black, and gray lines indicate the recombinant regions that belong to the genotype of LX4, CK/CH/LSC/99I, 4/91, Mass, Conn, Peafowl/GD/KQ6/2003, Taiwan-I, Lowa, and Georgia, respectively.

The recombination analysis showed that recombination events were found in the complete genome of 59 isolates (27.83%, 59/212), and 16 of them (7.55%, 16/212) were found in S1 gene (Figure 2 and Supplementary Tables 7, 8). The recombination regions were distributed throughout the entire genome. Based on the complete genome of 59 recombinant isolates, nsp3 and S genes were found to have the greatest number of recombination regions, with 28 and 26 recombination regions, respectively (Figure 2A). nsp16 (19 recombination regions) and N (17 recombination regions) genes were found to have the third and fourth greatest number of recombination regions. Based on S1 gene of 16 isolates, the midstream (600–700 bp) of S1 gene (9 recombination regions) was found to have the greatest number of recombination regions (Figure 2B). The downstream (1,500–1,611 bp) of S1 gene (8 recombination regions) was found to have the second greatest number of recombination regions. The detectable recombination breakpoint positions were located throughout the genome (Supplementary Figure 5A). A large number of breakpoints were observed in the 5′UTR, 1a, nsp3, nsp5, N, and 3′UTR. 5′UTR, nsp3, nsp16, S, and N genes were found to have the greatest number of transferred fragments (Table 1), which was consistent with the location and number of breakpoints in Supplementary Figure 5A. Moreover, the recombination breakpoint positions clustered in nsp3 and S genes. Thus, nsp3 and S genes were associated with the greatest number of transferred fragments. There was no breakpoint in the nsp8, nsp9, nsp10, and 3a regions. In addition, the detectable recombination breakpoint positions of S1 gene were shown in Supplementary Figure 5B. The recombination breakpoint positions were clustered within the midstream (600–700 bp) and downstream (1,500–1,611 bp) of S1 gene. In order to confirm the results of the RDP analysis, genomic sequence analyses of the complete genome of 59 isolates and S1 gene of 16 isolates were carried out by the Simplot software, and the results were consistent with those of the RDP analysis (Supplementary Figures 6, 7).

**TABLE 1:** Number of transferred fragments associated with individual areas of the genome for all of the isolates in China.

Genomic region	Number of fragments	% of total
5′UTR	13	7.47
nsp2	6	3.45
nsp3	29	16.67
nsp4	5	2.87
nsp5	8	4.60
nsp6	5	2.87
nsp7	3	1.72
nsp8	0	0.00
nsp9	0	0.00
nsp10	0	0.00
nsp11/12	8	4.60
nsp13	5	2.87
nsp14	7	4.02
nsp15	8	4.60
nsp16	10	5.75
Spike	28	16.09
3a	0	0.00
3b	1	0.57
Envelope	3	1.72
Membrane	6	3.45
5a	0	0.00
5b	6	3.45
Nucleocapsid	13	7.47
3′UTR	10	5.75

*UTR, untranslated region; nsp, non-structural protein.*

In addition, based on the complete genome sequences, a total of 52 of 59 (88.14%), 14 of 59 (23.73%), and 11 of 59 (18.64%) recombinants were likely from the LX4-type, 4/91-type, and Mass-type isolates, respectively (Supplementary Figure 5A and Supplementary Table 7). There were 20 recombinants formed by two major parents and two minor parent strains. There were four recombinants formed by three major parents and three minor parent strains. So multiple recombination events between the field and vaccine strains might occur in a total of 24 of the recombinant isolates (40.68%, 24/59) (Supplementary Table 7). Based on S1 gene, a total of 11 of 16 (68.75%) and 8 of 16 (50%) recombinants were likely from the LX4-type and 4/91-type isolates, respectively (Supplementary Figure 5B and Supplementary Table 8).

### Estimation of dN/dS Ratios on the S1, E, M, and N Proteins of Infectious Bronchitis Viruses

The results showed that the ratios of the mean substitution rates at the non-synonymous sites over those at the synonymous sites (dN/dS) for S1, S2, E, M, and N proteins of the 212 isolates were 0.301, 0.136, 0.225, 0.168, and 0.159, respectively (Supplementary Table 9). The dN/dS ratios for S1 protein of the LX4 type, New type, Taiwan-I type, Mass type, CK/CH/LSC99I type, 4/91 type, and LDT3-A type were 0.369, 0.358, 0.308, 0.307, 0.267, 0.239, and 0.174, respectively.

### Evolutionary Rates and Population Dynamics Analysis

**FIGURE 3:**
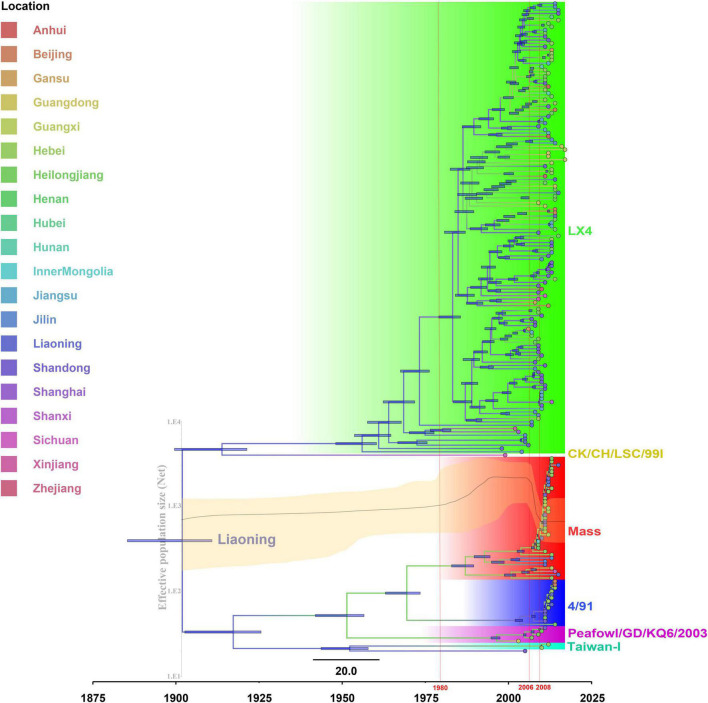
The maximum clade credibility tree and Bayesian skyline plot (BSP) of full-length genome sequences of infectious bronchitis viruses (IBVs) (see section “Materials and Methods” for further details). The maximum clade credibility (MCC) tree was constructed with 10% burn-in by Tree Annotator version 1.10.4 implemented in the BEAST software package. The color of the branches corresponds to geographical areas. A change in branch color indicates a time and geographical area change during the genotype’s evolutionary history. The scale bar represents the unit of time (year). Bayesian skyline plot (BSP) is superimposed to the tree in the same time-scale (*x*-axis). The *y*-axis represents the effective population size (log10 scale). The black line represents the effective population size through time, and the orange area around the line represents the 95% highest posterior density (HPD) of the estimates.

The temporal signal was assessed using TempEst software *via* root-to-tip regression analysis for the IBV isolates (correlation coefficient = 0.6576; *R*^2^ = 0.4325). In this test, the dataset was considered to have a relatively strong temporal signal for the sampling year. The evolution rate of IBVs was estimated to be 3.15 × 10^–3^ nucleotide substitutions/site/year (95% highest posterior density [HPD] 3.01E–3 to 3.29E–3). The IBVs in the last 35 years were classified into 6 main clusters by plotting the MCC tree (Figure 3). The estimated tMRCA of all the IBVs in China was the year 1901.94 (95% HPD [1885.44 to 1910.84]) found in Liaoning province, and the times of origin (95% HPD) of the major branch of viruses (LX4 type) in China was 1955.98 (95% HPD [1948.06 to 1960.31]) in Liaoning province also (Figure 3). A Bayesian skyline plot coalescent model was used to assess the reconstruction of population history. Figure 3 shows temporal changes in the effective population size of the viruses. Initially, the population diversity of the viruses showed a slow reduction before the 1980s. By 1980, the effective population size of the viruses had increased and undergone an upward trend after having fluctuated. The genetic diversity of the isolates increased rapidly and peaked until 2006 and a sudden and sharp decline in the effective population size was observed after 2006; thereafter, it persisted for 2 years. But since late 2008, the viral population has been rapidly rising again.

### Phylogeography Analyses of Infectious Bronchitis Virus in China

**FIGURE 4:**
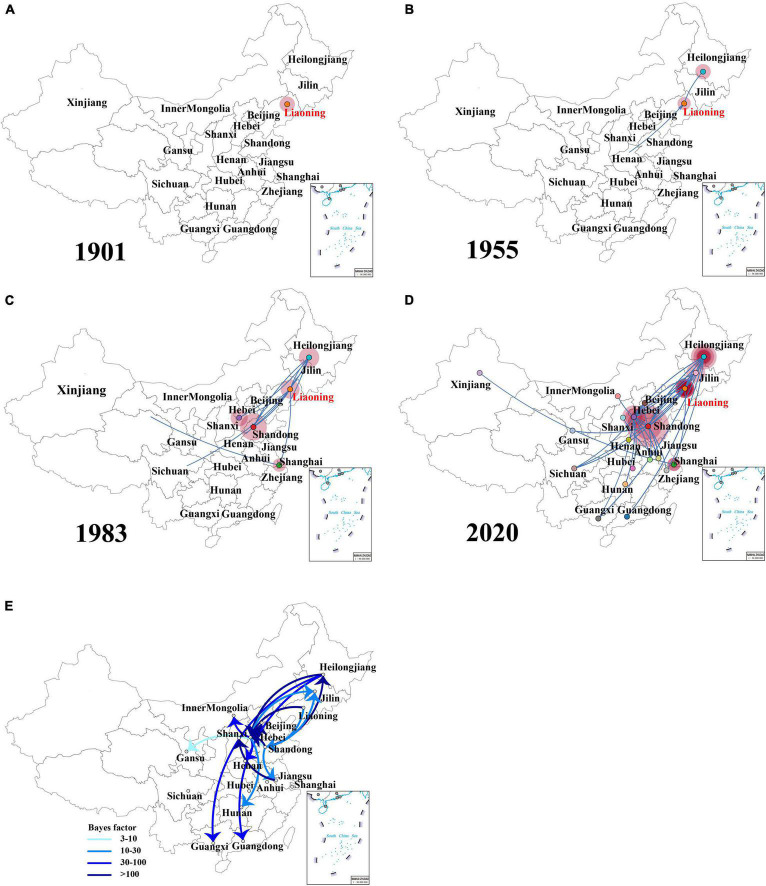
The dynamics analysis of geographical regions of the full-length genome sequences of field isolates from China sampled during the years 1985–2020 **(A–D)**. Spatiotemporal dynamics of the full-length genome gene sequences of infectious bronchitis virus (IBV) among 20 localities. Bayes factor (BF) test for significant non-zero rates and regional transmission analysis **(E)**. Curves show the among-country virus lineage transitions statistically supported with Bayes factor > 3 for all the IBV isolates in China. Curve colors represent corresponding statistical support (Bayes factor value) for each transition rate (inset).

The spatial dispersion patterns of IBVs in China during 1985–2020 were reconstructed. Figure 4 and Supplementary Table 10 show the major spread links of IBV. Generally, they spread between northern and southern China. The first transmission route was between the northern provinces Shandong, Hebei, and Beijing and the northeastern provinces Liaoning and Heilongjiang, with decisive support (BF > 100). The second transmission route was from Jiangsu to Shanxi with decisive support (BF > 100). The third transmission route was from Hebei to Henan and Inner Mongolia (BF > 30). The fourth transmission route was a long-distance movement event, notably from Heilongjiang to Guangdong and Guangxi (BF > 30). The fifth transmission route was from Hebei to Jilin, Hunan, Liaoning to Shandong, Shandong to Jilin, and Jiangsu (BF > 10). A long-distance movement event was from Shandong to Gansu (BF > 3). In accordance with the major spread links of IBV, Liaoning was the origin, which then propagated outwards in the early 1900s (Figure 4A). The directions of virus spread occurred predominantly from Liaoning to Heilongjiang in the late 1950s (Figure 4B), from the northeastern to northern in the early 1980s (Figure 4C), and from the northern to eastern and southern from 1980s to 2020 (Figure 4D). It is indicated that Shandong has been the epidemiological center of IBVs in China from the 1980s to 2020 (Figure 4E and Supplementary Table 10), based on the decisive spread routes from Shandong to Heilongjiang and Shandong to Hebei (BF > 1,000).

**FIGURE 5:**
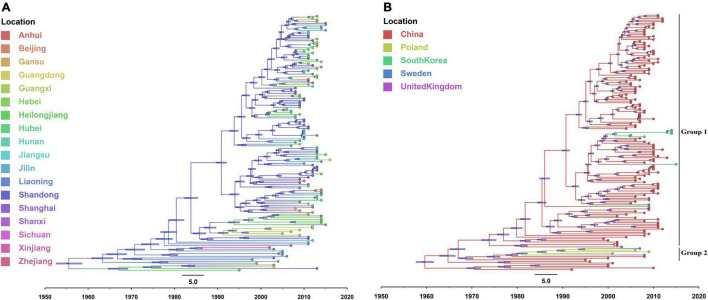
The maximum clade credibility tree of LX4-type full-length genome sequences of infectious bronchitis virus (IBV) isolates in China **(A)** and the world **(B)**. The color of the branches corresponds to geographical areas.

**FIGURE 6:**
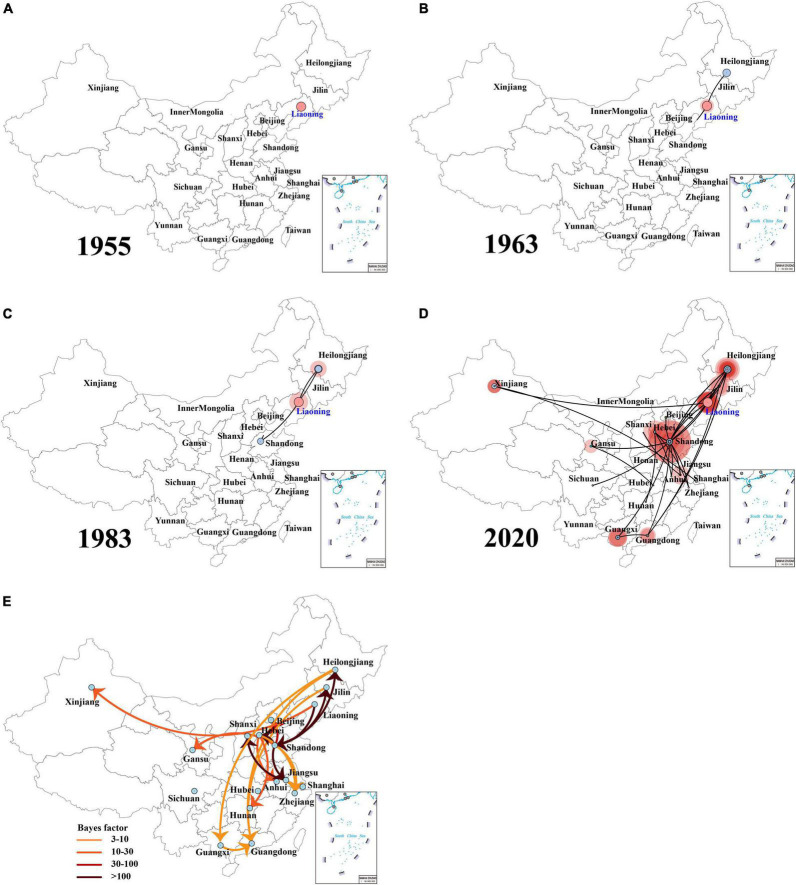
The dynamics analysis of geographical regions of the LX4 genotype isolates from China **(A–D)**. Spatiotemporal dynamics of the full-length genome gene sequences of LX4 genotype infectious bronchitis virus (IBV) among 18 localities. Bayes factor (BF) test for significant non-zero rates and regional transmission analysis **(E)**. Curves show the among-province virus lineage transitions statistically supported with Bayes factor > 3 for all the LX4 genotype IBV isolates in China.

An additional temporal signal of the dataset (152 LX4-type isolates in China based on Figure 1A and 15 LX4-type isolates in other countries based on the Supplementary Figure 3) was assessed. The results of the MCC tree (Figure 5A) showed that the sequences of the LX4-type IBV isolates in China were closely related to the early isolates of Liaoning in the late 1950s (correlation coefficient = 0.5945; *R*^2^ = 0.3534). The results of the BF value and the major spread links showed that the LX4-type isolates had multiple transmission routes in China (Figure 6 and Supplementary Table 11). The first transmission route was between Shandong and Liaoning, and Jilin and Heilongjiang (BF > 100). The second transmission route was between Shandong, Hebei, Shanxi, and Jiangsu (BF > 100). The third transmission route was between Shandong and Beijing (BF > 30). The fourth transmission route was between Shandong, Hebei, and Anhui, Zhejiang (BF > 3). The fifth transmission route was a long-distance movement event, notably from Heilongjiang, Jilin to Guangxi and Guangdong (BF > 3), Liaoning to Xinjiang, Shandong to Gansu, and Hebei to Hunan (BF > 10). Besides, a short-distance movement event was also observed from Guangxi to Guangdong (BF > 3). In accordance with the major spread links of the LX4 genotype, Liaoning was the origin of LX4 genotype IBV, which then propagated outwards in the late 1950s (Figures 3, 5A, 6). The directions of virus spread occurred predominantly from Liaoning to Heilongjiang in the early 1960s (Figure 6B), from the northeastern to northern regions in the early 1980s (Figure 6C), and from northern to eastern and southern regions (Figure 6D). Similarly, it is indicated that Shandong has been the epidemiological center of the LX4 genotype (Figure 6E and Supplementary Table 11), based on the decisive spread routes from Shandong to Heilongjiang, Shandong to Hebei, and Shandong to Jiangsu (BF > 1,000).

**FIGURE 7:**
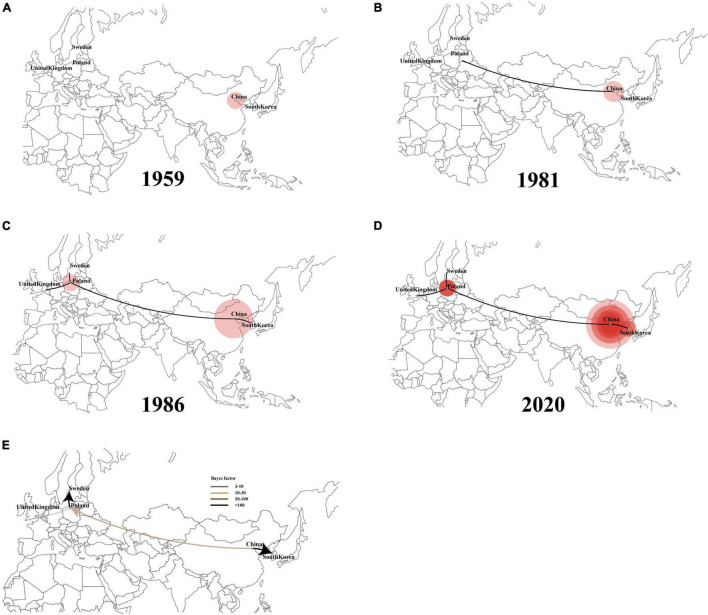
Spatiotemporal dynamics of LX4-type infectious bronchitis virus (IBV) isolates among different localities **(A–D)**. Bayes factor (BF) test for significant non-zero rates and regional transmission analysis **(E)**. Curves show the among-country virus lineage transitions statistically supported with Bayes factor > 3 for all the LX4 genotype IBV isolates in the world.

The results of the MCC tree (Figure 5B) showed that the sequences of Europe’s LX4-type isolates were closely related to the early isolates of China in the 1980s, which were in group 2. South Korea’s LX4-type isolates were closely related to the late isolates of China in the 1980s, which were in group 1 (correlation coefficient = 0.612; *R*^2^ = 0.3746). The results of BF value and the major spread links (Figure 7) showed that the LX4-type IBVs had two major transmission routes in the world: from China to Europe (Poland, Sweden, and so on) (BF > 10) and from China to South Korea (BF > 100).

## Discussion

The co-circulation of multiple IBV genotypes and the ongoing emergence of IBV variants have the greatest epidemiological challenges in China. In our study, a total of 6, 7, 7, 6, 6, and 5 genotypes were identified based on the sequences of the full-length genome and structural genes S1, S2, E, M, and N, respectively. According to the phylogenetic trees of both the whole genome and S1 gene, the top four dominant genotypes were the LX4 type, Mass type, 4/91 type, and Taiwan-I type, respectively. Therefore, our results showed that China was still in a state of coexistence of multiple genotypes with the LX4 type as the dominant genotype, which agreed with many previous descriptions (Liu et al., 2009; Zou et al., 2010; Li et al., 2012; Huang et al., 2019). We found that the LX4-type isolates have been dominant since 1999 in our study, which was earlier than those previous descriptions (Zhao et al., 2017; Jiang et al., 2018; Ren et al., 2020). The possible explanations are as follows: first, the isolates in our study were collected from 1985, while those in the previous studies were collected later than 1985. Second, the previous studies focused on the S1 gene sequences rather than the whole genome, but it is impossible to get a comprehensive understanding of the origins and evolutionary processes of those emerging viruses only by analyzing this small portion of the genome. In addition, the LX4 genotype was found to have the highest dN/dS ratios compared with other genotypes. High dN/dS ratios may promote the viral fitness in the infected hosts (Zhao et al., 2016). Thus, this is an explanation for why LX4 was the dominant genotype in recent years. Besides, we found that the Taiwan-I-type and New-type isolates showed an increasing trend since 2011 and 2015, respectively, in the country, which agreed with other previous studies (Xu et al., 2018; Fan et al., 2019; Huang et al., 2020), indicating that the commonly used commercial vaccines could not provide complete protection against these two genotype isolates. The development of new vaccines targeting Taiwan-I-type and New-type isolates should be of practical significance currently.

Recombination among coronaviruses could decrease mutational load, create genetic variation, and lead to the appearance of new isolates (Worobey and Holmes, 1999; Thor et al., 2011). Another study showed that some “hot spots” for recombination were in the regions of 1a (nsp2 and nsp3) and 1b (nsp16) of the genome and the upstream of the S gene based on the analysis of 25 America IBV isolates, and there were a high number of breakpoints in these three areas (Thor et al., 2011). However, our results showed that most of the breakpoints of recombination events may be located in nsp3 (1a region) and S gene (midstream and downstream) based on the 212 IBV isolates in China. Therefore, the breakpoints of recombination events of the IBV isolates in China were unique. Interestingly, no breakpoint in the regions of nsp8, nsp9, nsp10, and 3a was found. Areas that have the highest occurrence of recombination are located in regions of the genome that code for non-structural proteins 3 and the structural spike glycoprotein. A previous study showed that nsp3 encodes the protease PLP2, which is a type I interferon (IFN) antagonist and has a deubiquinating-like activity (Thor et al., 2011). Changes in the amino acid composition of the area in nsp3 could affect the ability of the virus to replicate in a variety of cell types (Thor et al., 2011). Moreover, the recombination in the area of nsp3 could alter the pathogenicity of the virus by modulating host cytokine expression (Eriksson et al., 2008; Thor et al., 2011). The spike glycoprotein exerts influence in attachment to host cell receptors, membrane fusion, and entry into the host cell (Thor et al., 2011). The spike glycoprotein has conformationally dependent epitopes that induce virus-neutralizing and serotype-specific antibodies (Cavanagh, 2007; Thor et al., 2011). Hence, the degree of recombination observed in the spike glycoprotein may be one of the principal mechanisms for generating genetic and antigenic diversity within IBV. The recombination regions within or near the S1 protein can lead to the emergence of new coronaviruses or new IBV serotypes (Thor et al., 2011). Our research showed that the recombination regions within S1 gene may be clustered in the midstream (600–700 bp) and downstream (1,500–1,611 bp) of the gene. The RBD was located in the midstream of S1 gene. The RBD was very important for the biological implications of genetic variation in circulating IBV genotypes (Bouwman et al., 2020). It was found that the amino acids 99–159 of RBD in the nephropathogenic IBV strain QX were required to establish kidney binding, and the reciprocal mutations in QX-RBD amino acids 110–112 could abolish kidney binding completely (Bouwman et al., 2020). Thus, more attention should be paid to these regions. In addition, the results of recombination analysis based on the whole genome and S1 gene would not be always consistent (Thor et al., 2011; Franzo et al., 2017). For example, based on the analysis result of recombination events in the complete genomes, there was a recombination region detected in the S1 region in EU637854 too. However, the *p*-value of S1 gene in EU637854 was lower than 10^–6^ but higher than 10^–12^ (10^–12^ < *p*-value < 10^–6^). We did not show such a result in Figure 2A.

China is the major producer of chickens worldwide. In spite of the widespread use of Mass type (H120, H52, Ma5, 28/86, M41, and W93), 4/91 type (NNA), LDT3-A, QXL87, and other vaccines, IB has been a continual problem in vaccinated flocks in China (Fan et al., 2018; Yan et al., 2019; Ren et al., 2020; Zhang et al., 2020). To obtain a comprehensive understanding of the epidemiological situation, evolutionary trend, and spatiotemporal diffusion of IBVs in China, a 35-year epidemiological analysis of a total of 212 IBV isolates from the years 1985 to 2020 on a national scale was performed here.

So far, there are three sequencing technologies, namely, Sanger dideoxy sequencing, next-generation sequencing (NGS), and third-generation sequencing (single molecular sequencing). The Sanger sequencing is read in longer sequences (800–1,000 bp), with high accuracy and ease of use, and its disadvantages were the limited capacity and relatively high costs (Heather and Chain, 2016). The low-fidelity polymerase errors could affect the result of genome sequences during the PCR, and the errors can be reduced by using a high-fidelity polymerase. The NGS allowed the sequencing of multiple genes, exomes, or genomes in a single experiment, which is far quicker and cheaper than the Sanger sequencing, but its operation processes require more steps and read in shorter sequences (400–500 bp) (Diekstra et al., 2015). The third-generation sequencing technology is developed rapidly with lower cost and more reads in long sequences, but it has more proportion of the wrong bases (Heather and Chain, 2016). According to the GenBank database, all the whole-genome sequences of 212 Chinese IBV isolate for analysis were obtained by Sanger dideoxy sequencing technology. Therefore, the sequencing method used for the sequences analyzed in the present study has little effect on the results.

Recent studies revealed that the 4/91-type strains have become an important gene donor to variants in the genetic evolution of IBV in China (Zhao et al., 2016; Fan et al., 2019; Xu et al., 2019; Huang et al., 2020). Our studies showed that the percentage of 4/91-type recombinants (23.73 and 50% recombinants based on the full-length genome and S1, respectively) may be higher than that of Mass-type recombinants (18.64 and 12.50% recombinants based on the full-length genome and S1, respectively) in the present study, which agreed with our previous reports (Mo et al., 2013; Fan et al., 2019). In the previous study, we analyzed several reasons why the 4/91-type strains were more prone to recombination than other vaccine-type strains (Fan et al., 2019). Given the infrequent prevalence of the 4/91-type isolates since 2015, it is recommended that the 4/91-type vaccine is to be used sparingly or with caution, but continued monitoring is needed. In addition, we found that multiple recombination events between field and vaccine strains may have occurred in the 24 recombinant isolates based on the complete genomes of the 212 Chinese isolates, suggesting the complexity of the recombination mechanism of IBV. Our results also suggest that genome-wide recombination analysis, not just S1 gene recombination analysis, should be performed in order to have a comprehensive understanding of recombination events of IBV isolates.

Bayesian phylodynamic models have not been widely used for disease surveillance in the poultry industry. However, they have been used widely to analyze the evolution and spatial spreading of SARS-CoV (Rest and Mindell, 2003), MERS-CoV (Breban et al., 2013), and SARS-CoV-2 (Li et al., 2020). In our study, the abovementioned models were used for analyzing the domestic history of IBVs in China to serve as a real-time early warning monitoring system and help the managers to make the correct decisions to reduce the risk of infection. According to our results, the Liaoning isolates were at the root of the tree, with the highest root state posterior probability in the 1900s. Thus, Liaoning was the origin of IBV in China in 1901. To our knowledge, this is the first report referring to the origin of the Chinese IBV on both the exact time and place by the Bayesian phylodynamic models. However, the inferred past geographical locations are heavily limited by the input data. All sequences were from provinces in China, and thus the inferred origin of the virus in the dataset would undoubtedly be one of the provinces of China. In China, Liaoning used to be one of the major provinces of poultry production and has a close relationship with live bird trading with other provinces. In addition, based on the MCC tree, the LX4-type isolates were first found in Liaoning in the late 1950s and then spread to other provinces. Franzo et al. (2017) found that the QX genotype (LX4 type, GI-19) originated from China in the late 1970s, and the GI-19 lineage circulated within the country until it migrated to Europe in the late 1990s, based on the analysis of S1 gene of total of 807 strains from 18 countries by plotting the MCC tree. The first LX4-type isolate (QX strain) was reported in Qingdao, Shandong province, in 1996 (Wang et al., 1997). The first case reported in Qingdao in 1996 did not mean that the virus first appeared in that time and area. Zhao et al. found that many LX4-type IBVs disappeared before 2005 because they were not “fit” for adaptation in chickens, but those “fit” LX4-type IBVs continued to evolve and have become widespread and predominant in commercial poultry after 2005 (Zhao et al., 2017). The LX4 (GI-19) lineage had circulated for a long time in China before its recognition as a distinct genotype in 1996 (Franzo et al., 2017). Hence, our result first demonstrated that Liaoning province was the origin of LX4-type IBV in the late 1950s. Besides, in order to get a full picture of the virus evolution, we try to divide the alignment into several recombination-free regions such as S and E genes and do a separate analysis on each of them instead. However, different genomic regions have different evolutionary histories. Therefore, in order to obtain comprehensive genetic information and molecular mechanism of variation of IBV isolates and mapped to the S gene (the greatest number of recombination regions and the very strong signal), we excluded the complete genome of 16 isolates, which were the potential recombinant sequences in S gene, and used the dataset to analyze the evolution and spatial spreading of viruses.

A previous report showed that the avian influenza virus’s transmission occurred along the poultry industry trade routes (Yang et al., 2020). Interestingly, a similar phenomenon appeared in our study based on the IBV trade routes. In our study, five trade routes for both IBV and LX4-type isolates in China were strongly indicated with high BF values. Moreover, the first transmission route (from Shandong to Heilongjiang and Jilin), the second one (from Jiangsu to Shanxi, from Shandong to Jiangsu and Hebei), the fifth one (from the northeastern province Heilongjiang and Jilin to the southern provinces Guangxi and Guangdong), and the long-distance movement events (from the northern province Shandong to the northwestern province Gansu and from Hebei to the southern province Hunan) of the LX4-type isolates were similar to those of the IBVs in China. The northern region including Shandong and Liaoning provinces is the biggest region of white chicken production in China with annually 1.8 and 0.8 billion birds, respectively, while Guangxi and Guangdong had the biggest production of the local breeds of chickens (1.9 billion birds) in 2019 (Wei, 2019; Deng et al., 2021). Hence, the five transmission routes were basically consistent with poultry industry trade routes.

In order to provide more targeted strategies for the prevention and control of the disease, we employed a Bayesian phylogeographic approach. Based on the IBV spread routes, all IBVs especially the LX4-type isolates in China exhibited evidence of a continuous process of geographic spread. Nowadays, northeastern, northern, and southern regions of China were the most frequent places of the viral output and input, indicating that these three areas have been the epicenter of the LX4 genotype in China. A previous report showed that the intensity of the poultry trade among locations and the migration of wild birds among locations in China were positively associated with avian influenza virus lineage spread, and the distances along the road network in China were strongly inversely associated with viral lineage spread (Yang et al., 2020). We found that the IBVs (including LX4 type) spread routes and that the live poultry trade network was positively associated with the viral lineage spread. Hence, the IBVs in the Chinese mainland were mainly spread through the transport and trade of live birds, while the long-distance spreads, which were from the northeastern Liaoning province to northwestern Xinjiang province and the northeastern provinces Heilongjiang, and Jilin to the southern provinces Guangxi and Guangdong, were more likely through migratory wild birds. These data on mode of geographic spread would play a critical role in the prevention and control of IB.

To further investigate the source of the LX4-type isolates in the world, we also conducted phylogeographic inference. The full-length genome sequences of the early LX4-type isolates during 2004–2018 in other countries were found to be closely related to the early isolates in China during 1995–2016, as they were at the lower level of the node in the tree, which means that the LX4-type isolates were likely to have originated from China. In addition, tMRCA of the earliest LX4-type isolates was estimated to be in the late 1950s according to the Bayesian analyses. The LX4-type isolates had spread from China to Europe in the early 1980s and spread from China to South Korea in the late 1980s based on the Bayesian analyses. Of course, the inferred transmissions of LX4-type IBV might not be direct, but the virus may have gone unobserved through multiple countries before entering Europe/South Korea. Other researchers reported that the LX4-type strain was from South Korea based on the analysis of S1 genotype, the time of isolating, and the geographical distance between Shandong province and South Korea (Liu et al., 2006; Huang et al., 2019). However, analysis of S1 gene alone had a limitation. The Bayesian phylodynamic models have been used widely to analyze the evolution and spatial spreading of SARS-CoV-2 (Li et al., 2020). Hence, a thorough and comprehensive Bayesian analysis using as many strains across the world with the entire genome sequences could contribute to a greater understanding of the spread of IBV. Of course, our research also had some data limitations. For example, the number of samples was inhomogeneous in different countries.

The generation, maintenance, and distribution of genetic variation of the virus can be affected by a sudden change in population size (Gao et al., 2019). Indeed, our demographic analyses reveal that IBV populations in China had been small before the 1980s but have undergone expansion since the 1980s, possibly associated with a capacity expansion of chicken in China after China adopted the reform and opening-up policy. However, a sudden and sharp decrease in genetic diversity was observed after 2006, probably due to an outbreak of the H5N1 virus in migratory waterfowl in Qinghai in 2005 and spread to other areas (Chen et al., 2005), which had brought substantial damage to the poultry industry. A large number of farms were closed due to being affected by the pandemic avian influenza (Wei, 2019). At that time, the inter-provincial trade of live poultry was banned, which was in accordance with our result that the IBV population declined in size from 2006. However, since late 2008, a sharp increase was observed in viral population size. There may be two main reasons to explain this phenomenon. First, new farms were increasing because of the successful control of the avian influenza outbreak. The inter-provinces’ live poultry trade was allowed. Second, the LX4-type isolates had become the predominant IBVs since 2008 based on the analysis of S1 gene (Mo et al., 2013; Fan et al., 2019), and the implementation of the current vaccines could not provide good protection against the circulating strains. Other studies indicated that the change of viral population size had a strong association with the vaccine administration/withdrawal (Franzo et al., 2014, 2016). However, the protection provided by the new commercial LX4-type vaccine (QXL87) on the market against the LX4-type isolates has not been studied yet, which needs further investigation.

## Conclusion

In conclusion, Chinese IBV was still in a state of coexistence of multiple genotypes, the LX4 type with the highest dN/dS ratios has been the most dominant genotype since 1999, and the Taiwan-I type and New type showed an increasing trend recently. A high frequency of recombination and multiple recombination events have occurred in the complete genome, and the 4/91-type strains were more prone to be involved in the recombination. The Chinese IBV originated from Liaoning in the early 1900s and has three main clusters: the northeastern cluster, the northern cluster, and the southern cluster. In particular, the LX4 type originated from Liaoning in the late 1950s and had multiple transmission routes in China plus two major transmission routes to other countries. Shandong has been the epidemiological center of IBVs (including LX4 type) in China. These data extend our insight into the evolution and spread of IBV in China and provide more targeted strategies for the prevention and control of IB in China.

## Data Availability Statement

The data analyzed during this study were available within the article and its supporting information.

## Author Contributions

WF analyzed the data and wrote the manuscript. JC and YZ contributed to the experiment. PW and MM provided the funding of research and reviewed and approved the final manuscript. All authors participated in this study, read the final manuscript, and approved it for submission.

## Conflict of Interest

The authors declare that the research was conducted in the absence of any commercial or financial relationships that could be construed as a potential conflict of interest.

## Publisher’s Note

All claims expressed in this article are solely those of the authors and do not necessarily represent those of their affiliated organizations, or those of the publisher, the editors and the reviewers. Any product that may be evaluated in this article, or claim that may be made by its manufacturer, is not guaranteed or endorsed by the publisher.

## Footnotes

1
https://www.ncbi.nlm.nih.gov

2
http://www.resdc.cn/data.aspx?dataid=243

3
http://mafft.cbrc.jp/alignment/software/

4
https://github.com/ddarriba/jmodeltest2

5
http://www.datamonkey.org/

6
http://beast.community/index.html

7
http://tree.bio.ed.ac.uk

## Supplementary Material

Supplementary Figure 1 Distribution of 212 IBV isolates in China. Distribution is presented as the number of isolates per province or autonomous city (zone), from most cases (45, dark red) to no case (0, white).

Supplementary Figure 2 The percentages of different genotypes based on the complete genome sequence **(A)**, S1 **(B)**, S2 **(C)**, E **(D)**, M **(E)**, and N **(F)** of IBV strains in different years. There was only one IBV strain (GX-C) isolated during 1985–1998, which showed considerable low homology with the above other genotypes and belonged to a separate group.

Supplementary Figure 3 Phylogenetic trees of the complete genome of 68 IBVs from other countries and the 33 IBV reference strains. Phylogenetic trees were constructed with the maximum likelihood method using RaxML software. Green represent LX4 genotype. The green solid dots represent 15 LX4 genotype IBV isolates.

Supplementary Figure 4 Alignment of the deduced amino acid sequences of the S1 subunit of the spike protein of 212 IBV isolates and the references M41, H120, 4/91, QXL87, LX4, and LDT3-A strains. Amino acid sequences encoding the three hypervariable regions are signed in red line. The minimal receptor-binding domains corresponding to the most N-terminal residues 19–272 of the spike protein of IBV M41 are boxed, and the critical amino acids 70–74 are highlighted in red. KM213963: the isolate of CK/CH/XDC-2/2013; KP118886: the isolate of ck/CH/LSD/111235; KC008600: the isolate of GX-C; HQ848267: the isolate of GX-YL5; HQ850618: the isolate of GX-YL9; KF411041: the isolate of CK/CH/LGX/091109; KP118894: the isolate of ck/CH/LGD/090907; KC013541: the isolate of Ck/CH/LGD/120723; KC119407: the isolate of Ck/CH/LGD/120724; JF893452: the isolate of YN; MK644086: the isolate of E160_YN.

Supplementary Figure 5 The vaccine strain H120 was used as the reference strain. **(A)** The virus genome of the coronavirus avian infectious bronchitis showing the locations of 5′ and 3′ UTRs and the coding regions. Recombination breakpoints identified across the full-length genome. The putative recombinants were listed in the key, with the colored circles matching each corresponding breakpoint. The beginning breakpoint was highlighted in  circles and the ending breakpoint was highlighted in  circles from 5′ to 3′ UTRs in every recombinant isolate. **(B)** Recombination breakpoints identified across the S1 gene. The beginning breakpoint was highlighted in  circle and the ending breakpoint was highlighted in  circle in every recombinant isolate.

Supplementary Figure 6 Similarity plots of the full-length genome of 59 IBV isolates created with SimPlot version 3.5.1. A 200-bp window with a 20-bp step was used.

Supplementary Figure 7 Similarity plots of the S1 structural gene region of 16 IBV isolates created with SimPlot version 3.5.1. A 200-bp window with a 20-bp step was used.

## References

[B1] Baele G. Li W. L. S. Drummond A. J. Suchard M. A. Lemey P. (2012). Accurate model selection of relaxed molecular clocks in Bayesian phylogenetics. *Mol. Bio. Evol.* 30 239–243. 10.1093/molbev/mss243 23090976 PMC3548314

[B2] Bande F. Arshad S. S. Omar A. R. Hair-Bejo M. Mahmuda A. Nair V. (2017). Global distributions and strain diversity of avian infectious bronchitis virus: a review. *Anim Health Res. Rev.* 18 70–83. 10.1017/S1466252317000044 28776490

[B3] Bielejec F. Baele G. Vrancken B. Suchard M. A. Rambaut A. Lemey P. (2016). SpreaD3: interactive visualization of spatiotemporal history and trait evolutionary processes. *Mol. Bio. Evol.* 33 2167–2169. 10.1093/molbev/msw082 27189542 PMC6398721

[B4] Bouwman K. M. Parsons L. M. Berends A. J. de Vries R. P. Cipollo J. F. Verheije M. H. (2020). Three amino acid changes in avian coronavirus spike protein allow binding to kidney tissue. *J. Virol.* 94 e1363–19. 10.1128/JVI.01363-19 31694947 PMC6955270

[B5] Breban R. Riou J. Fontanet A. (2013). Interhuman transmissibility of Middle East respiratory syndrome coronavirus: estimation of pandemic risk. *Lancet* 382 694–699. 10.1016/S0140-6736(13)61492-0 23831141 PMC7159280

[B6] Cavanagh D. (2007). Coronavirus avian infectious bronchitis virus. *Vet Res.* 38 281–297. 10.1051/vetres:2006055 17296157

[B7] Cavanagh D. Davis P. J. Cook J. K. Li D. Kant A. Koch G. (1992). Location of the amino acid differences in the S1 spike glycoprotein subunit of closely related serotypes of infectious bronchitis virus. *Avian Pathol.* 21 33–43. 10.1080/03079459208418816 18670913

[B8] Chen H. Smith G. Zhang S. Y. Qin K. Wang J. Li K. S. (2005). H5N1 virus outbreak in migratory waterfowl. *Nature* 436 191–192. 10.1038/nature03974 16007072

[B9] Darriba D. Taboada G. L. Doallo R. Posada D. (2012). jModelTest 2: more models, new heuristics and parallel computing. *Nat. Methods* 9:772. 10.1038/nmeth.2109 22847109 PMC4594756

[B10] Deng Q. Shi M. Li Q. Wang P. Li M. Wang W. (2021). Analysis of the evolution and transmission dynamics of the field MDV in China during the years 1995-2020, indicating the emergence of a unique cluster with the molecular characteristics of vv+MDV that has become endemic in southern China. *Transboundary Emerg. Dis.* 68 3574–3587. 10.1111/tbed.13965 33354907

[B11] Diekstra A. Bosgoed E. Rikken A. van Lier B. Kamsteeg E. J. Tychon M. (2015). Translating sanger-based routine DNA diagnostics into generic massive parallel ion semiconductor sequencing. *Clin. Chem.* 61 154–162. 10.1373/clinchem.2014.225250 25274553

[B12] Drosten C. Gunther S. Preiser W. van der W. S. Brodt H. R. Becker S. (2003). Identification of a novel coronavirus in patients with severe acute respiratory syndrome. *N. Engl. J. Med.* 348 1967–1976. 10.1056/NEJMoa030747 12690091

[B13] Eriksson K. K. Cervantes B. L. Ludewig B. Thiel V. (2008). Mouse hepatitis virus Liver pathology is dependent on ADP-ribose-1”-phosphatase, a viral function conserved in the alpha-Like supergroup. *J. Virol.* 82 12325–12334. 10.1128/JVI.02082-08 18922871 PMC2593347

[B14] Fan W. Li H. He Y. Tang N. Zhang L. Wang H. (2018). Immune protection conferred by three commonly used commercial live attenuated vaccines against the prevalent local strains of avian infectious bronchitis virus in southern China. *J. Vet. Med. Sci.* 80 1438–1444. 10.1292/jvms.18-0249 30022779 PMC6160892

[B15] Fan W. Tang N. Dong Z. Chen J. Zhang W. Zhao C. (2019). Genetic analysis of avian coronavirus infectious bronchitis virus in yellow chickens in southern China over the past decade: revealing the changes of genetic diversity, dominant genotypes, and selection pressure. *Viruses* 11:898. 10.3390/v11100898 31561498 PMC6833030

[B16] Finger P. F. Pepe M. S. Dummer L. A. Magalhaes C. G. de Castro C. C. de Oliveira H. S. (2018). Combined use of ELISA and Western blot with recombinant N protein is a powerful tool for the immunodiagnosis of avian infectious bronchitis. *Virol. J.* 15:189. 10.1186/s12985-018-1096-2 30541588 PMC6292099

[B17] Franzo G. Massi P. Tucciarone C. M. Barbieri I. Tosi G. Fiorentini L. (2017). Think globally, act locally: phylodynamic reconstruction of infectious bronchitis virus (IBV) QX genotype (GI-19 lineage) reveals different population dynamics and spreading patterns when evaluated on different epidemiological scales. *PLoS One* 12:e0184401. 10.1371/journal.pone.0184401 28880958 PMC5589226

[B18] Franzo G. Naylor C. J. Lupini C. Drigo M. Catelli E. Listorti V. (2014). Continued use of IBV 793B vaccine needs reassessment after its withdrawal led to the genotype’s disappearance. *Vaccine* 32 6765–6767. 10.1016/j.vaccine.2014.10.006 25446828 PMC7172084

[B19] Franzo G. Tucciarone C. M. Blanco A. Nofrarias M. Biarnes M. Cortey M. (2016). Effect of different vaccination strategies on IBV QX population dynamics and clinical outbreaks. *Vaccine* 34 5670–5676. 10.1016/j.vaccine.2016.09.014 27670071 PMC7173296

[B20] Gao F. Liu X. Du Z. Hou H. Wang X. Wang F. (2019). Bayesian phylodynamic analysis reveals the dispersal patterns of tobacco mosaic virus in China. *Virology* 528 110–117. 10.1016/j.virol.2018.12.001 30594790

[B21] Heather J. M. Chain B. (2016). The sequence of sequencers: the history of sequencing DNA. *Genomics* 107 1–8. 10.1016/j.ygeno.2015.11.003 26554401 PMC4727787

[B22] Huang M. Zhang Y. Xue C. Cao Y. (2019). To meet the growing challenge: research of avian infectious bronchitis in China. *Microbiol. China.* 46 1837–1849. 10.13344/j.microbiol.china.180898

[B23] Huang M. Zou C. Liu Y. Han Z. Xue C. Cao Y. (2020). A novel low virulent respiratory infectious bronchitis virus originating from the recombination of QX, TW and 4/91 genotype strains in China. *Vet. Microbiol.* 242:108579. 10.1016/j.vetmic.2020.108579 32122588 PMC7111478

[B24] Ignjatovic J. Sapats S. (2005). Identification of previously unknown antigenic epitopes on the S and N proteins of avian infectious bronchitis virus. *Arch. Virol.* 150 1813–1831. 10.1007/s00705-005-0541-x 15868095 PMC7087300

[B25] Jackwood M. W. Boynton T. O. Hilt D. A. McKinley E. T. Kissinger J. C. Paterson A. H. (2010). Emergence of a group 3 coronavirus through recombination. *Virology* 398 98–108. 10.1016/j.virol.2009.11.044 20022075 PMC7111905

[B26] Jackwood M. W. Lee D. (2017). Different evolutionary trajectories of vaccine-controlled and non-controlled avian infectious bronchitis viruses in commercial poultry. *PLoS One* 12:e0176709. 10.1371/journal.pone.0176709 28472110 PMC5417570

[B27] Jiang L. Han Z. Chen Y. Zhao W. Sun J. Zhao Y. (2018). Characterization of the complete genome, antigenicity, pathogenicity, tissue tropism, and shedding of a recombinant avian infectious bronchitis virus with a ck/CH/LJL/140901-like backbone and an S2 fragment from a 4/91-like virus. *Virus Res.* 244 99–109. 10.1016/j.virusres.2017.11.007 29141204 PMC7114561

[B28] Katoh K. Standley D. M. (2013). MAFFT Multiple sequence alignment software version 7: improvements in performance and usability. *Mol. Biol. Evol.* 30 772–780. 10.1093/molbev/mst010 23329690 PMC3603318

[B29] Lauer S. A. Grantz K. H. Bi Q. Jones F. K. Zheng Q. Meredith H. R. (2020). The incubation period of coronavirus disease 2019 (COVID-19) from publicly reported confirmed cases: estimation and application. *Ann. Intern. Med.* 172 577–582. 10.7326/M20-0504 32150748 PMC7081172

[B30] Li M. Wang X. Wei P. Chen Q. Wei Z. Mo M. (2012). Serotype and genotype diversity of infectious bronchitis viruses isolated during 1985-2008 in Guangxi. *China. Arch. Virol.* 157 467–474. 10.1007/s00705-011-1206-6 22198411

[B31] Li X. Zai J. Zhao Q. Nie Q. Li Y. Foley B. T. (2020). Evolutionary history, potential intermediate animal host, and cross-species analyses of SARS-CoV-2. *J. Med. Virol.* 92 602–611. 10.1002/jmv.25731 32104911 PMC7228310

[B32] Liang J. Fang S. Yuan Q. Huang M. Chen R. Fung T. (2019). N-Linked glycosylation of the membrane protein ectodomain regulates infectious bronchitis virus-induced ER stress response, apoptosis and pathogenesis. *Virology* 531 48–56. 10.1016/j.virol.2019.02.017 30852271 PMC7112112

[B33] Lin S. Chen H. (2017). Infectious bronchitis virus variants: molecular analysis and pathogenicity investigation. *Int. J. Mol. Sci.* 18:2030. 10.3390/ijms18102030 28937583 PMC5666712

[B34] Liu S. Zhang Q. Chen J. Han Z. Liu X. Feng L. (2006). Genetic diversity of avian infectious bronchitis coronavirus strains isolated in China between 1995 and 2004. *Arch. Virol.* 151 1133–1148. 10.1007/s00705-005-0695-6 16397751 PMC7086752

[B35] Liu S. Zhang X. Wang Y. Li C. Han Z. Shao Y. (2009). Molecular characterization and pathogenicity of infectious bronchitis coronaviruses: complicated evolution and epidemiology in China caused by cocirculation of multiple types of infectious bronchitis coronaviruses. *Intervirology* 52 223–234. 10.1159/000227134 19590226

[B36] Martin D. P. (2009). Recombination detection and analysis using RDP3. *Methods Mol. Biol.* 537 185–205. 10.1007/978-1-59745-251-9_9 19378145

[B37] Mo M. Li M. Huang B. Fan W. Wei P. Wei T. (2013). Molecular characterization of major structural protein genes of avian coronavirus infectious bronchitis virus isolates in southern china. *Viruses* 5 3007–3020. 10.3390/v5123007 24304696 PMC3967158

[B38] Molenaar R. J. Dijkman R. de Wit J. J. (2020). Characterization of infectious bronchitis virus D181, a new serotype (GII-2). *Avian Pathol.* 49 243–250. 10.1080/03079457.2020.1713987 31951468

[B39] Nguyen L. T. Schmidt H. A. von Haeseler A. Minh B. Q. (2015). IQ-TREE: a fast and effective stochastic algorithm for estimating maximum-likelihood phylogenies. *Mol. Biol. Evol.* 32 268–274. 10.1093/molbev/msu300 25371430 PMC4271533

[B40] Rambaut A. Drummond A. J. Xie D. Baele G. Suchard M. A. (2018). Posterior summarization in Bayesian phylogenetics using Tracer 1.7. *Syst. Biol.* 67 901–904. 10.1093/sysbio/syy032 29718447 PMC6101584

[B41] Rambaut A. Lam T. T. Max, Carvalho L. Pybus O. G. (2016). Exploring the temporal structure of heterochronous sequences using TempEst (formerly Path-O-Gen). *Virus Evol.* 2:vew007. 10.1093/ve/vew007 27774300 PMC4989882

[B42] Ren G. Liu F. Huang M. Li L. Shang H. Liang M. (2020). Pathogenicity of a QX-like avian infectious bronchitis virus isolated in China. *Poult. Sci.* 99 111–118. 10.3382/ps/pez568 32416792 PMC7111634

[B43] Rest J. S. Mindell D. P. (2003). SARS associated coronavirus has a recombinant polymerase and coronaviruses have a history of host-shifting. *Infect. Genet. Evol.* 3 219–225. 10.1016/j.meegid.2003.08.001 14522185 PMC7129878

[B44] Suchard M. A. Lemey P. Baele G. Ayres D. L. Drummond A. J. Rambaut A. (2018). Bayesian phylogenetic and phylodynamic data integration using BEAST 1.10. *Virus Evol.* 4:vey016. 10.1093/ve/vey016 29942656 PMC6007674

[B45] Tamura K. Stecher G. Peterson D. Filipski A. Kumar S. (2013). MEGA6: molecular evolutionary genetics analysis version 6.0. *Mol. Biol. Evol.* 30 2725–2729. 10.1093/molbev/mst197 24132122 PMC3840312

[B46] Thor S. W. Hilt D. A. Kissinger J. C. Paterson A. H. Jackwood M. W. (2011). Recombination in avian gamma-coronavirus infectious bronchitis virus. *Viruses* 3 1777–1799. 10.3390/v3091777 21994806 PMC3187689

[B47] Valastro V. Holmes E. C. Britton P. Fusaro A. Jackwood M. W. Cattoli G. (2016). S1 gene-based phylogeny of infectious bronchitis virus: an attempt to harmonize virus classification. *Infect. Genet. Evol.* 39 349–364. 10.1016/j.meegid.2016.02.015 26883378 PMC7172980

[B48] Wang Y. Zhang Z. Wang Y. Fan G. Jiang Y. Liu X. (1997). A preliminary report on the study of glandular stomach type infectious bronchitis of chickens. *Chin. J. Anim. Quarant.* 14 6–8.

[B49] Wei P. (2019). Challenge and development opportunity of quality chicken industry in China: a review about 9 issues on the topic. *China Poult.* 41 1–6. 10.16372/j.issn.1004-6364.2019.11.001

[B50] Worobey M. Holmes E. C. (1999). Evolutionary aspects of recombination in RNA viruses. *J. Gen. Virol.* 80 2535–2543. 10.1099/0022-1317-80-10-2535 10573145

[B51] Wu Q. Lin Z. Qian K. Shao H. Ye J. Qin A. (2019). Peptides with 16R in S2 protein showed broad reactions with sera against different types of infectious bronchitis viruses. *Vet. Microbiol.* 236:108391. 10.1016/j.vetmic.2019.108391 31500728 PMC7117385

[B52] Xu L. Han Z. Jiang L. Sun J. Zhao Y. Liu S. (2018). Genetic diversity of avian infectious bronchitis virus in China in recent years. *Infect. Genet. Evol.* 66 82–94. 10.1016/j.meegid.2018.09.018 30244092 PMC7185438

[B53] Xu L. Ren M. Sheng J. Ma T. Han Z. Zhao Y. (2019). Genetic and biological characteristics of four novel recombinant avian infectious bronchitis viruses isolated in China. *Virus Res.* 263 87–97. 10.1016/j.virusres.2019.01.007 30641197 PMC7185608

[B54] Yan S. Sun Y. Huang X. Jia W. Xie D. Zhang G. (2019). Molecular characteristics and pathogenicity analysis of QX-like avian infectious bronchitis virus isolated in China in 2017 and 2018. *Poult. Sci.* 98 5336–5341. 10.3382/ps/pez351 31222258 PMC7107289

[B55] Yang Q. Zhao X. Lemey P. Suchard M. A. Bi Y. Shi W. (2020). Assessing the role of live poultry trade in community-structured transmission of avian influenza in China. *Proc. Natl. Acad. Sci. U. S. A.* 117 5949–5954. 10.1073/pnas.1906954117 32123088 PMC7084072

[B56] Yuan Y. Zhang Z. He Y. Fan W. Dong Z. Zhang L. (2018). Protection against virulent infectious bronchitis virus challenge conferred by a recombinant baculovirus co-expressing S1 and N proteins. *Viruses* 10:347. 10.3390/v10070347 29954092 PMC6071288

[B57] Zaki A. M. van Boheemen S. Bestebroer T. M. Osterhaus A. D. M. E. Fouchier R. A. M. (2012). Isolation of a novel coronavirus from a man with pneumonia in Saudi Arabia. *N. Engl. J. Med.* 367 1814–1820. 10.1056/NEJMoa1211721 23075143

[B58] Zhang X. Deng T. Lu J. Zhao P. Chen L. Qian M. (2020). Molecular characterization of variant infectious bronchitis virus in China, 2019: implications for control programmes. *Transbound. Emerg. Dis.* 67 1349–1355. 10.1111/tbed.13477 31943814 PMC7228276

[B59] Zhao W. Gao M. Xu Q. Xu Y. Zhao Y. Chen Y. (2017). Origin and evolution of LX4 genotype infectious bronchitis coronavirus in China. *Vet. Microbiol.* 198 9–16. 10.1016/j.vetmic.2016.11.014 28062013 PMC7117135

[B60] Zhao Y. Zhang H. Zhao J. Zhong Q. Jin J. Zhang G. (2016). Evolution of infectious bronchitis virus in China over the past two decades. *J. Gen. Virol.* 97 1566–1574. 10.1099/jgv.0.000464 27008625 PMC7079583

[B61] Zou N. Zhao F. Wang Y. Liu P. Cao S. Wen X. (2010). Genetic analysis revealed LX4 genotype strains of avian infectious bronchitis virus became predominant in recent years in Sichuan area, China. *Virus Genes.* 41 202–209. 10.1007/s11262-010-0500-9 20556502 PMC7089292

