# Supplementary material for: Phylogenetic and Spatiotemporal Analyses of the Complete Genome Sequences of Avian Coronavirus Infectious Bronchitis Virus in China During 1985–2020: Revealing Coexistence of Multiple Transmission Chains and the Origin of LX4-Type Virus
Source: Front Microbiol. 2022 Apr 4;13:693196. doi: 10.3389/fmicb.2022.693196 (PMC9013971; doi:10.3389/fmicb.2022.693196)
Supplement: Supplementary Figure 1 — Distribution of 212 IBV isolates in China. Distribution is presented as the number of isolates per province or autonomous city (zone), from most cases (45, dark red) to no case (0, white). [file Data_Sheet_1.ZIP › Supplementary material/Supplementary Figure 4.pdf]

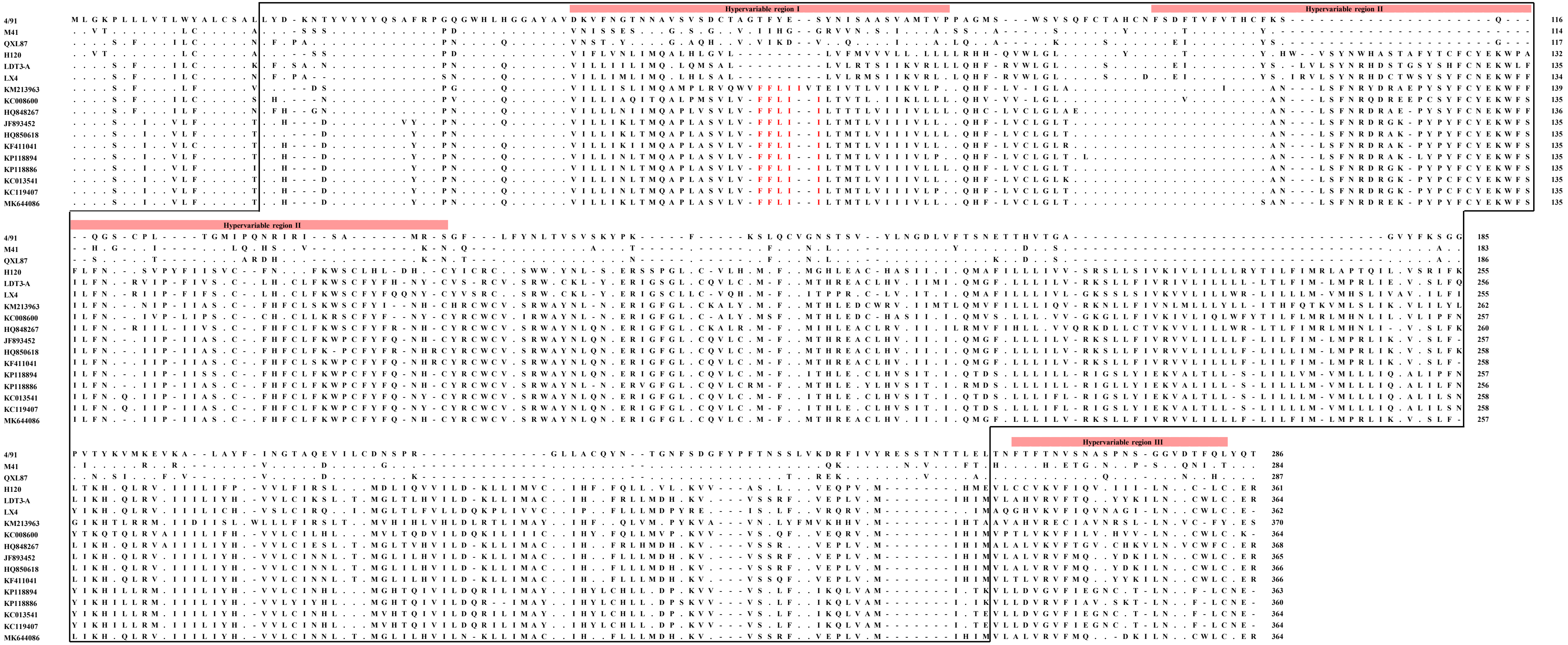

Hypervariable region I
Hypervariable region II
Hypervariable region III
4/91
M41
QXL87
H120
LDT3-A
LX4
KM213963
KC008600
HQ848267
JF893452
HQ850618
KF411041
KP118894
KP118886
KC013541
KC119407
MK644086
116 114 117 132 135 134 139 135 136 135 135 135 135 135 135 135 135
185 183 186 255 256 255 262 257 260 257 258 258 257 256 258 258 257
286 284 287 361 364 362 370 364 368 365 366 366 363 360 364 364 364
