# Supplementary material for: Phylogenetic and Spatiotemporal Analyses of the Complete Genome Sequences of Avian Coronavirus Infectious Bronchitis Virus in China During 1985–2020: Revealing Coexistence of Multiple Transmission Chains and the Origin of LX4-Type Virus
Source: Front Microbiol. 2022 Apr 4;13:693196. doi: 10.3389/fmicb.2022.693196 (PMC9013971; doi:10.3389/fmicb.2022.693196)
Supplement: Supplementary Figure 1 — Distribution of 212 IBV isolates in China. Distribution is presented as the number of isolates per province or autonomous city (zone), from most cases (45, dark red) to no case (0, white). [file Data_Sheet_1.ZIP › Supplementary material/Supplementary Table 1.pdf]

**Supplementary Table 1.** Background information of IBV isolates in China used in this study.

| IBV isolates | Years of isolation | Province of origin | Accession numbers | Host | Sequencing technology |
|---|---|---|---|---|---|
| ck/CH/LAH/120721 | 2012 | Anhui | KX348117 | Chicken | Sanger |
| ck/CH/LAH/120907 | 2012 | Anhui | KX348115 | Chicken | Sanger |
| ahysx-1 | 2016 | Anhui | MK142676 | Chicken | Sanger |
| ck/CH/LBJ/140402 | 2014 | Beijing | KP118882 | Chicken | Sanger |
| ck/CH/LBJ/140413 | 2014 | Beijing | KP118881 | Chicken | Sanger |
| ck/CH/LBJ/140511 | 2014 | Beijing | KX364295 | Chicken | Sanger |
| ck/CH/LBJ/140528 | 2014 | Beijing | KX364294 | Chicken | Sanger |
| ck/CH/LBJ/120481 | 2012 | Beijing | KX389094 | Chicken | Sanger |
| ck/CH/LBJ/140514 | 2014 | Beijing | KX348116 | Chicken | Sanger |
| ck/CH/LGS/131148 | 2013 | Gansu | KX302874 | Chicken | Sanger |
| ck/CH/LGS/08I | 2008 | Gansu | KX185058 | Chicken | Sanger |
| ck/CH/LGS/06I | 2006 | Gansu | KX219792 | Chicken | Sanger |
| CK/CH/GD/GZ14 | 2014 | Guangdong | KT946798 | Yellow-chicken | Sanger |
| ck/CH/LGD/090907 | 2009 | Guangdong | KP118894 | Yellow-chicken | Sanger |
| Ck/CH/LGD/120724 | 2012 | Guangdong | KC119407 | Yellow-chicken | Sanger |
| CK/CH/GD/QY16 | 2016 | Guangdong | MG197727 | Yellow-chicken | Sanger |
| CK/CH/GD/GDTS13 | 2016 | Guangdong | MN197549 | Yellow-chicken | Sanger |
| CK-CH-GX-YL17_2017 | 2017 | Guangdong | MK329221 | Yellow-chicken | Sanger |
| CK/CH/GD/HY16 | 2017 | Guangdong | MK309398 | Yellow-chicken | Sanger |
| Ck/CH/LGD/120723 | 2012 | Guangdong | KC013541 | Yellow-chicken | Sanger |
| GX-C | 1985 | Guangxi | KC008600 | Yellow-chicken | Sanger |
| GX-NN09032 | 2009 | Guangxi | JX897900 | Yellow-chicken | Sanger |
| GX-YL9 | 2007 | Guangxi | HQ850618 | Yellow-chicken | Sanger |
| GX-YL5 | 2005 | Guangxi | HQ848267 | Yellow-chicken | Sanger |
| ck/CH/LGX/111119 | 2011 | Guangxi | KX640829 | Yellow-chicken | Sanger |
| ck/CH/LGX/130530 | 2013 | Guangxi | KP343691 | Yellow-chicken | Sanger |
| CK/CH/LGX/091109 | 2009 | Guangxi | KF411041 | Yellow-chicken | Sanger |
| ck/CH/LHB/130578 | 2013 | Hebei | KP118890 | Chicken | Sanger |
| ck/CH/LHB/130575 | 2013 | Hebei | KP118889 | Chicken | Sanger |
| ck/CH/LHB/140532 | 2014 | Hebei | KP118887 | Chicken | Sanger |
| ck/CH/LHB/121041 | 2012 | Hebei | KP118883 | Chicken | Sanger |
| ck/CH/LHB/130927 | 2013 | Hebei | KP118880 | Chicken | Sanger |
| ck/CH/LHB/130630 | 2013 | Hebei | KP036504 | Chicken | Sanger |

| | | | | | |
|---|---|---|---|---|---|
| ck/CH/LHB/121010 | 2012 | Hebei | KP036503 | Chicken | Sanger |
| ck/CH/LHB/131143 | 2013 | Hebei | KJ425502 | Chicken | Sanger |
| ck/CH/LHB/131142 | 2013 | Hebei | KJ425501 | Chicken | Sanger |
| ck/CH/LHB/131132 | 2013 | Hebei | KJ425500 | Chicken | Sanger |
| ck/CH/LHB/131118 | 2013 | Hebei | KJ425499 | Chicken | Sanger |
| ck/CH/LHB/130642 | 2013 | Hebei | KJ425498 | Chicken | Sanger |
| ck/CH/LHB/130598 | 2013 | Hebei | KJ425497 | Chicken | Sanger |
| ck/CH/LHB/130573 | 2013 | Hebei | KJ425496 | Chicken | Sanger |
| ck/CH/LHB/121040 | 2012 | Hebei | KJ425495 | Chicken | Sanger |
| ck/CH/LHB/121024 | 2012 | Hebei | KJ425494 | Chicken | Sanger |
| ck/CH/LHB/120749 | 2012 | Hebei | KJ425493 | Chicken | Sanger |
| ck/CH/LHB/120403 | 2012 | Hebei | KJ425492 | Chicken | Sanger |
| ck/CH/LHB/111268 | 2011 | Hebei | KJ425491 | Chicken | Sanger |
| ck/CH/LHB/111232 | 2011 | Hebei | KJ425490 | Chicken | Sanger |
| ck/CH/LHB/111172 | 2011 | Hebei | KJ425489 | Chicken | Sanger |
| ck/CH/LHB/110825 | 2011 | Hebei | KJ425488 | Chicken | Sanger |
| ck/CH/LHB/110526 | 2011 | Hebei | KJ425487 | Chicken | Sanger |
| ck/CH/LHB/100801 | 2010 | Hebei | JF330898 | Chicken | Sanger |
| ck/CH/LHB/110123 | 2011 | Hebei | KX400753 | Chicken | Sanger |
| ck/CH/LHB/090921 | 2009 | Hebei | KX236016 | Chicken | Sanger |
| ck/CH/LHB/120497 | 2014 | Hebei | KX236014 | Chicken | Sanger |
| ck/CH/LHB/150619 | 2015 | Hebei | KX252781 | Chicken | Sanger |
| ck/CH/LHB/131144 | 2013 | Hebei | KX252776 | Chicken | Sanger |
| ck/CH/LHB/130628 | 2013 | Hebei | KX252775 | Chicken | Sanger |
| ck/CH/LHB/130589 | 2013 | Hebei | KX252774 | Chicken | Sanger |
| ck/CH/LHB/140542 | 2014 | Hebei | KX252773 | Chicken | Sanger |
| ck/CH/LHB/120402 | 2012 | Hebei | KX247130 | Chicken | Sanger |
| ck/CH/LHB/130337 | 2013 | Hebei | KX247129 | Chicken | Sanger |
| ck/CH/LHB/130569 | 2013 | Hebei | KX247128 | Chicken | Sanger |
| ck/CH/LHB/111190 | 2011 | Hebei | KX247127 | Chicken | Sanger |
| ck/CH/LHB/111168 | 2011 | Hebei | KX219793 | Chicken | Sanger |
| ck/CH/LHB/121042 | 2012 | Hebei | MG448607 | Chicken | Sanger |
| ck/CH/LHLJ/140906 | 2014 | Heilongjiang | KP036502 | Chicken | Sanger |
| ck/CH/LHLJ/111246 | 2011 | Heilongjiang | KP118891 | Chicken | Sanger |
| ck/CH/LHLJ/07VII | 2007 | Heilongjiang | JF274479 | Chicken | Sanger |
| ck/CH/LHLJ/131216 | 2013 | Heilongjiang | KJ425507 | Chicken | Sanger |
| ck/CH/LHLJ/111050 | 2011 | Heilongjiang | KJ425506 | Chicken | Sanger |
| ck/CH/LHLJ/110310 | 2011 | Heilongjiang | KJ425505 | Chicken | Sanger |

| | | | | | |
|---|---|---|---|---|---|
| ck/CH/LHLJ/091205 | 2009 | Heilongjiang | KJ425504 | Chicken | Sanger |
| ck/CH/LHLJ/090908 | 2009 | Heilongjiang | KJ425503 | Chicken | Sanger |
| ck/CH/LHLJ/100902 | 2010 | Heilongjiang | JF828980 | Chicken | Sanger |
| ck/CH/LHLJ/130622 | 2013 | Heilongjiang | KX434788 | Chicken | Sanger |
| ck/CH/LHLJ/99I | 1999 | Heilongjiang | KX375808 | Chicken | Sanger |
| ck/CH/LHLJ/110836 | 2011 | Heilongjiang | KX375807 | Chicken | Sanger |
| ck/CH/LHLJ/110673 | 2011 | Heilongjiang | KX375806 | Chicken | Sanger |
| ck/CH/LHLJ/121219 | 2012 | Heilongjiang | KX372249 | Chicken | Sanger |
| ck/CH/LHLJ/130705 | 2013 | Heilongjiang | KX275393 | Chicken | Sanger |
| ck/CH/LHLJ/110943 | 2011 | Heilongjiang | KX275392 | Chicken | Sanger |
| ck/CH/LHLJ/140927 | 2014 | Heilongjiang | KX275391 | Chicken | Sanger |
| ck/CH/LHLJ/141103 | 2014 | Heilongjiang | KX275390 | Chicken | Sanger |
| ck/CH/LHLJ/130822 | 2013 | Heilongjiang | KX302870 | Chicken | Sanger |
| ck/CH/LHLJ/090806 | 2009 | Heilongjiang | KX302869 | Chicken | Sanger |
| ck/CH/LHLJ/130744 | 2013 | Heilongjiang | KX302868 | Chicken | Sanger |
| ck/CH/LHLJ/150701 | 2015 | Heilongjiang | KX236004 | Chicken | Sanger |
| ck/CH/LHLJ/090515 | 2009 | Heilongjiang | KX252790 | Chicken | Sanger |
| ck/CH/LHLJ/090605 | 2009 | Heilongjiang | KX252789 | Chicken | Sanger |
| ck/CH/LHLJ/08-6 | 2008 | Heilongjiang | KX252788 | Chicken | Sanger |
| ck/CH/LHLJ/090510 | 2009 | Heilongjiang | KX252783 | Chicken | Sanger |
| ck/CH/LHLJ/07I | 2007 | Heilongjiang | KX219791 | Chicken | Sanger |
| CK/CH/LHLJ/140901 | 2014 | Heilongjiang | KP790146 | Chicken | Sanger |
| CK/CH/LHLJ/141105 | 2014 | Heilongjiang | KP790145 | Chicken | Sanger |
| CK/CH/LHLJ/140756 | 2014 | Heilongjiang | KP790144 | Chicken | Sanger |
| ck/CH/LHLJ/111043 | 2011 | Heilongjiang | KP868572 | Chicken | Sanger |
| ck/CH/LHN/090909 | 2009 | Henan | KJ425508 | Chicken | Sanger |
| ck/CH/LHuB/131123 | 2013 | Hubei | KX302871 | Chicken | Sanger |
| ck/CH/LHN/120338 | 2012 | Hunan | KX259253 | Chicken | Sanger |
| ck/CH/LHN/101211 | 2010 | Hunan | KX259248 | Chicken | Sanger |
| ck/CH/IBTZ/2012 | 2012 | Jiangsu | KF663559 | Chicken | Sanger |
| ck/CH/LJS/111210 | 2011 | Jiangsu | KX434790 | Chicken | Sanger |
| ck/CH/LJS/07V | 2007 | Jiangsu | KX375805 | Chicken | Sanger |
| ck/CH/LJS/120848 | 2012 | Jiangsu | KX259257 | Chicken | Sanger |
| ck/CH/LJS/120552 | 2012 | Jiangsu | KX259256 | Chicken | Sanger |
| ck/CH/LJS/111111 | 2011 | Jiangsu | KX259255 | Chicken | Sanger |
| ck/CH/LJS/110439 | 2011 | Jiangsu | KX259252 | Chicken | Sanger |
| ck/CH/LJS/101237 | 2010 | Jiangsu | KX259251 | Chicken | Sanger |
| ck/CH/LJS/101113 | 2010 | Jiangsu | KX259250 | Chicken | Sanger |

| | | | | | |
|---|---|---|---|---|---|
| ck/CH/LJL/100512 | 2010 | Jiangsu | KX302875 | Chicken | Sanger |
| ck/CH/LJS/131102 | 2013 | Jiangsu | KX302865 | Chicken | Sanger |
| ck/CH/LJS/101111 | 2010 | Jiangsu | KX302862 | Chicken | Sanger |
| ck/CH/LJS/131049 | 2013 | Jiangsu | KX302861 | Chicken | Sanger |
| ck/CH/LJS/101109 | 2010 | Jiangsu | KX219794 | Chicken | Sanger |
| ck/CH/LJL/130925 | 2013 | Jilin | KP036505 | Chicken | Sanger |
| ck/CH/LJL/121059 | 2012 | Jilin | KJ425509 | Chicken | Sanger |
| ck/CH/LJL/110302 | 2011 | Jilin | KC136209 | Chicken | Sanger |
| ck/CH/LJL/131006 | 2013 | Jilin | KX364297 | Chicken | Sanger |
| ck/CH/LJL/08-9 | 2008 | Jilin | KX364296 | Chicken | Sanger |
| ck/CH/LJL/07III | 2007 | Jilin | KX364293 | Chicken | Sanger |
| ck/CH/LJL/101150 | 2010 | Jilin | KX364290 | Chicken | Sanger |
| ck/CH/LJL/140530 | 2014 | Jilin | KX434789 | Chicken | Sanger |
| ck/CH/LJL/140734 | 2014 | Jilin | KX425847 | Chicken | Sanger |
| ck/CH/LJL/090330 | 2009 | Jilin | KX259249 | Chicken | Sanger |
| ck/CH/LJL/140924 | 2014 | Jilin | KX302873 | Chicken | Sanger |
| ck/CH/LJL/150430 | 2015 | Jilin | KX302867 | Chicken | Sanger |
| ck/CH/LJL/04I | 2004 | Jilin | KX302866 | Chicken | Sanger |
| ck/CH/LJL/08-1 | 2008 | Jilin | KX236005 | Chicken | Sanger |
| ck/CH/LJL/05I | 2005 | Jilin | KX252778 | Chicken | Sanger |
| CK/CH/LJL/130908 | 2013 | Jilin | KP868573 | Chicken | Sanger |
| ck/CH/LJL/111054 | 2011 | Jilin | KC506155 | Chicken | Sanger |
| ck/CH/LLN/130101 | 2013 | Liaoning | KP118892 | Chicken | Sanger |
| ck/CH/LLN/130102 | 2013 | Liaoning | KP118888 | Chicken | Sanger |
| ck/CH/LDL/120557 | 2012 | Liaoning | KJ425486 | Chicken | Sanger |
| ck/CH/LDL/110931 | 2011 | Liaoning | KJ425485 | Chicken | Sanger |
| ck/CH/LDL/101212 | 2010 | Liaoning | JF828981 | Chicken | Sanger |
| ck/CH/LDL/091022 | 2009 | Liaoning | JX195175 | Chicken | Sanger |
| ck/CH/LDL/05II | 2005 | Liaoning | KX364298 | Chicken | Sanger |
| ck/CH/LDL/140709 | 2014 | Liaoning | KX236015 | Chicken | Sanger |
| ck/CH/LDL/130325 | 2013 | Liaoning | KX236008 | Chicken | Sanger |
| ck/CH/LLN/131040 | 2013 | Liaoning | KX252787 | Chicken | Sanger |
| ck/CH/LLN/120605 | 2012 | Liaoning | KX252786 | Chicken | Sanger |
| ck/CH/LLN/090910 | 2009 | Liaoning | KX252784 | Chicken | Sanger |
| ck/CH/LLN/07I | 2007 | Liaoning | KX252782 | Chicken | Sanger |
| ck/CH/LLN/090312 | 2009 | Liaoning | KX252780 | Chicken | Sanger |
| ck/CH/LLN/06I | 2006 | Liaoning | KX252779 | Chicken | Sanger |
| ck/CH/LDL/05III | 2005 | Liaoning | KX348114 | Chicken | Sanger |

| | | | | | |
|---|---|---|---|---|---|
| ckCHLDL150434-II | 2015 | Liaoning | KT736032 | Chicken | Sanger |
| ck/CH/LDL/150434-I | 2015 | Liaoning | KT736031 | Chicken | Sanger |
| CK/CH/LDL/140520 | 2014 | Liaoning | KP790143 | Chicken | Sanger |
| CK/CH/LLN/111169 | 2011 | Liaoning | KF411040 | Chicken | Sanger |
| ck/CH/LLN/98I | 1998 | Liaoning | KX252791 | Chicken | Sanger |
| ck/CH/LDL/150434-III | 2015 | Liaoning | KX077987 | Chicken | Sanger |
| ck/CH/LNM/091017 | 2009 | Inner Mongolia | JF330899 | Chicken | Sanger |
| CK/CH/LSD/05I | 2005 | Shandong | EU637854 | Chicken | Sanger |
| CK/CH/SD/121220 | 2012 | Shandong | KJ128295 | Chicken | Sanger |
| CK/CH/XDC-2/2013 | 2013 | Shandong | KM213963 | Chicken | Sanger |
| ck/CH/LSD/110410 | 2011 | Shandong | KP118893 | Chicken | Sanger |
| ck/CH/LSD/111235 | 2011 | Shandong | KP118886 | Chicken | Sanger |
| ck/CH/LSD/110857 | 2011 | Shandong | KP118885 | Chicken | Sanger |
| ck/CH/LSD/110851 | 2011 | Shandong | KP118884 | Chicken | Sanger |
| SDZB0808 | 2008 | Shandong | KF853202 | Chicken | Sanger |
| CK/CH/SD09/005 | 2009 | Shandong | KF668605 | Chicken | Sanger |
| ck/CH/LSD/1112150 | 2011 | Shandong | KJ435286 | Chicken | Sanger |
| ck/CH/LSD/121228 | 2012 | Shandong | KJ435285 | Chicken | Sanger |
| ck/CH/LSD/111241 | 2011 | Shandong | KJ435284 | Chicken | Sanger |
| ck/CH/LSD/111219 | 2011 | Shandong | KJ435283 | Chicken | Sanger |
| ck/CH/LSD/110726 | 2011 | Shandong | KJ425512 | Chicken | Sanger |
| ck/CH/LSD/110529 | 2011 | Shandong | KJ425511 | Chicken | Sanger |
| ck/CH/LSD/110505 | 2011 | Shandong | KJ425510 | Chicken | Sanger |
| ck/CH/LSD/111218 | 2011 | Shandong | KX364300 | Chicken | Sanger |
| ck/CH/LSD/101223 | 2010 | Shandong | KX364299 | Chicken | Sanger |
| ck/CH/LSD/110739 | 2011 | Shandong | KX364292 | Chicken | Sanger |
| ck/CH/LSD/110409 | 2011 | Shandong | KX364291 | Chicken | Sanger |
| ck/CH/LSD/130205 | 2013 | Shandong | KX372250 | Chicken | Sanger |
| ck/CH/LSD/090314 | 2009 | Shandong | KX259254 | Chicken | Sanger |
| ck/CH/LSD08-8 | 2008 | Shandong | KX275394 | Chicken | Sanger |
| ck/CH/LSD/101115 | 2010 | Shandong | KX302872 | Chicken | Sanger |
| ck/CH/LSD/09091 | 2009 | Shandong | KX302864 | Chicken | Sanger |
| ck/CH/LSD/090334 | 2009 | Shandong | KX302863 | Chicken | Sanger |
| ck/CH/LSD08-7 | 2008 | Shandong | KX302860 | Chicken | Sanger |
| ck/CH/LSD/120742 | 2012 | Shandong | KX236013 | Chicken | Sanger |
| ck/CH/LSD/091003 | 2009 | Shandong | KX236012 | Chicken | Sanger |
| ck/CH/LSD/091203 | 2009 | Shandong | KX236011 | Chicken | Sanger |
| ck/CH/LSD/100412 | 2010 | Shandong | KX236010 | Chicken | Sanger |

| | | | | | |
|---|---|---|---|---|---|
| ck/CH/LSD/100408 | 2010 | Shandong | KX236007 | Chicken | Sanger |
| ck/CH/LSD/100305 | 2010 | Shandong | KX236006 | Chicken | Sanger |
| ck/CH/LSD/111025 | 2011 | Shandong | KX236003 | Chicken | Sanger |
| ck/CH/LSD/111037 | 2011 | Shandong | KX236002 | Chicken | Sanger |
| ck/CH/LSD/03I | 2003 | Shandong | KX236001 | Chicken | Sanger |
| ck/CH/LSD/110912 | 2011 | Shandong | KX236000 | Chicken | Sanger |
| ck/CH/LSD/100311 | 2010 | Shandong | KX252785 | Chicken | Sanger |
| ck/CH/LSD/07I | 2007 | Shandong | KX252777 | Chicken | Sanger |
| ck/CH/LSD/120437 | 2012 | Shandong | KX219801 | Chicken | Sanger |
| ck/CH/LSD/130611 | 2013 | Shandong | KX219800 | Chicken | Sanger |
| ck/CH/LSD/130211 | 2013 | Shandong | KX219799 | Chicken | Sanger |
| ck/CH/LSD/120913 | 2012 | Shandong | KX219798 | Chicken | Sanger |
| ck/CH/LSD/150311 | 2015 | Shandong | KX219795 | Chicken | Sanger |
| SDIB821/2012 | 2012 | Shandong | KF574761 | Chicken | Sanger |
| ck/CH/LSHH/03II | 2003 | Shanghai | KX252772 | Chicken | Sanger |
| ck/CH/LSX/130132 | 2013 | Shanxi | KX236009 | Chicken | Sanger |
| Sczy3 | 2009 | Sichuan | JF732903 | Chicken | Sanger |
| ck/CH/SCYB/140913 | 2014 | Sichuan | KU356856 | Chicken | Sanger |
| SAIBK2 | 2011 | Sichuan | KU317090 | Chicken | Sanger |
| 3575/08 | 2008 | Taiwan | KX266757 | Chicken | Sanger |
| ck/CH/LXJ/02I | 2002 | Xinjiang | KX219797 | Chicken | Sanger |
| ck/CH/LXJ/111265 | 2011 | Xinjiang | KX219796 | Chicken | Sanger |
| YN | 2005 | Yunnan | JF893452 | Yellow-chicken | Sanger |
| ck/CH/YNSL/160501 | 2016 | Yunnan | MN096598 | Yellow-chicken | Sanger |
| E160_YN | 2018 | Yunnan | MK644086 | Yellow-chicken | Sanger |
| YX10 | 2010 | Zhejiang | JX840411 | Chicken | Sanger |
| ck/CH/LZJ/111113 | 2011 | Zhejiang | JX195176 | Chicken | Sanger |
