# Supplementary material for: Phylogenetic and Spatiotemporal Analyses of the Complete Genome Sequences of Avian Coronavirus Infectious Bronchitis Virus in China During 1985–2020: Revealing Coexistence of Multiple Transmission Chains and the Origin of LX4-Type Virus
Source: Front Microbiol. 2022 Apr 4;13:693196. doi: 10.3389/fmicb.2022.693196 (PMC9013971; doi:10.3389/fmicb.2022.693196)
Supplement: Supplementary Figure 1 — Distribution of 212 IBV isolates in China. Distribution is presented as the number of isolates per province or autonomous city (zone), from most cases (45, dark red) to no case (0, white). [file Data_Sheet_1.ZIP › Supplementary material/Supplementary Table 6.doc]

**Supplementary Table 6.** Candidate phylodynamic models for IBV

| Molecular clock | Population prior | AICM |
| --- | --- | --- |
| Strict | Constant Size | 832299.496 |
|  | **Bayesian Skyline** | 829650.504 |
| Uncorrelated Lognormal | Constant Size | 1021608.15 |
|  | Bayesian Skyline | 1997378.622 |

Note: All the models are convergent (ESS > 200). The best models are highlighted in boldface.
