# Supplementary material for: Phylogenetic and Spatiotemporal Analyses of the Complete Genome Sequences of Avian Coronavirus Infectious Bronchitis Virus in China During 1985–2020: Revealing Coexistence of Multiple Transmission Chains and the Origin of LX4-Type Virus
Source: Front Microbiol. 2022 Apr 4;13:693196. doi: 10.3389/fmicb.2022.693196 (PMC9013971; doi:10.3389/fmicb.2022.693196)
Supplement: Supplementary Figure 1 — Distribution of 212 IBV isolates in China. Distribution is presented as the number of isolates per province or autonomous city (zone), from most cases (45, dark red) to no case (0, white). [file Data_Sheet_1.ZIP › Supplementary material/Supplementary Table 7.doc]

**Supplementary Table 7.** Genetic recombination events of full-length genome sequences of IBV isolates detected by RDP 4 software.

| **Potential**  **recombinant** | **Breakpoints** | | **Genes ^b^** | **Major parent ^c^**  **(similarity)** | **Minor parent ^d^**  **(similarity)** | **Detection method ^a^** |
| --- | --- | --- | --- | --- | --- | --- |
|  | **Beginning** | **Ending** |  |  |  |  |
| KC008600_GX-C_1985_Guangxi | 14484 | 17333 | Nsp11/12, Nsp13, Nsp14 | CK/CH/LSC/99I (CK/CH/LSC/99I-type)  (90.9%) | KJ435284_ck/CH/LSD/111241_2011_Shandong (Mass-type)  (98.4%) | RDP, GENECONV, MaxChi, Chimera, 3Seq |
|  | 18916 | 20327 | Nsp15, Nsp16 | KX219798_ck/CH/LSD/120913_2012_Shandong (LX4-type)  (unknown) **^e^** | JX897900_GX-NN09032_2009_Guangxi (LX4-type)  (99.7%) | RDP, GENECONV, MaxChi, Chimera, 3Seq |
| KX252791_ck/CH/LLN/98I_1998_Liaoning | 14383 | 16023 | Nsp11/12,  Nsp13 | KX434788_ck/CH/LHLJ/130622_2013_Heilongjiang (LX4-type)  (unknown) | KX236015_ck/CH/LDL/140709_2014_Liaoning (LX4-type)  (98.3%) | RDP, GENECONV, MaxChi, Chimera, 3Seq |
|  | 24642 | 26958 | Membrane, 4b,  4c, 5a, 5b,  Nucleocapsid | KP118886_ck/CH/LSD/111235_2011_Shandong (LX4-type)  (93.3%) | Arkansas_DPI  (Conn-type)  (99.5%) | RDP, GENECONV, Bootscan, MaxChi, Chimera, 3Seq |
| KX375808_ck/CH/LHLJ/99I_1999_Heilongjiang | 2885 | 3994 | Nsp3 | LX4 (LX4-type)  (92.2%) | KX364294_ck/CH/LBJ/140528_2014_Beijing (LX4-type)  (93.8%) | RDP, GENECONV, MaxChi, Chimera, 3Seq |
|  | 9543 | 10729 | Nsp5, Nsp6,  Nsp7 | KX364292_ck/CH/LSD/110739_2011_Shandong (LX4-type)  (92.4%) | JF893452_YN_2005_Yunnan (CK/CH/LSC/99I-type)  (96.5%) | RDP, GENECONV, Bootscan, MaxChi, Chimera, 3Seq |
|  | 26648 | 27535 | Nucleocapsid, 6b, 3'UTR | KX252772_ck/CH/LSHH/03II_2003_Shanghai (LX4-type)  (93.6%) | KP036504_ck/CH/LHB/130630_2013_Hebei (4/91-type)  (97.5%) | RDP, GENECONV, Bootscan, MaxChi, Chimera, 3Seq |
| KY799582_ck/CH/LSC/99I_1999_Sichuan | 2679 | 4358 | Nsp3 | ck/CH/LHLJ/95I  (LX4-type)  (unknown) | KX236006_ck/CH/LSD/100305_2010_Shandong (LX4-type)  (96.2%) | RDP, GENECONV, Bootscan, MaxChi, Chimera, 3Seq |
| KX219797_ck/CH/LXJ/02I_2002_Xinjiang | 1 | 2766 | 5'UTR, Nsp2,  Nsp3 | KX364295_ck/CH/LBJ/140511_2014_Beijing (LX4-type)  (94.9%) | CK/CH/LSC/99I  (CK/CH/LSC/99I-type)  (95.4%) | RDP, GENECONV, Bootscan, MaxChi, Chimera, 3Seq |
|  | 12989 | 15882 | Nsp11/12,  Nsp13 | KX302861_ck/CH/LJS/131049_2013_Jiangsu (LX4-type)  (unknown) | KP790143_CK/CH/LDL/140520_2014_Liaoning (LX4-type)  (98.7%) | RDP, GENECONV, MaxChi, Chimera, 3Seq |
|  | 18762 | 20179 | Nsp15, Nsp16 | KX434788_ck/CH/LHLJ/130622_2013_Heilongjiang (LX4-type)  (unknown) | KX252780_ck/CH/LLN/090312_2009_Liaoning (LX4-type)  (98.4%) | RDP, GENECONV, MaxChi, Chimera, 3Seq |
| KX236001_ck/CH/LSD/03I_2003_Shandong | 9966 | 24302 | Nsp6, Nsp7, Nsp8, Nsp9, Nsp10, Nsp11/12, Nsp13, Nsp14, Nsp15, Nsp16, Spike, 3a, 3b, Envelope | KP118893_ck/CH/LSD/110410_2011_Shandong (4/91-type)  (87.5%) | KX236010_ck/CH/LSD/100412_2010_Shandong (LX4-type)  (96.8%) | RDP, GENECONV, Bootscan, MaxChi, Chimera |
|  | 25745 | 27038 | 5b, Nucleocapsid | KJ425508_ck/CH/LHN/090909_2009_Henan (Mass-type)  (97.7%) | KJ435286_ck/CH/LSD/1112150_2011_Shandong  (Peafowl/GD/KQ6/2003-type)  (98.2%) | RDP, GENECONV, Bootscan, MaxChi, Chimera, 3Seq |
| KX302866_ck/CH/LJL/04I_2004_Jilin | 9553 | 10788 | Nsp5, Nsp6,  Nsp7 | KX364292_ck/CH/LSD/110739_2011_Shandong (LX4-type)  (93.4%) | JF893452_YN_2005_Yunnan (CK/CH/LSC/99I-type)  (96.5%) | RDP, GENECONV, Bootscan, MaxChi, Chimera, 3Seq |
| KX252778_ck/CH/LJL/05I_2005_Jilin | 1 | 2393 | 5'UTR, Nsp2 | KX252780_ck/CH/LLN/090312_2009_Liaoning (LX4-type)  (96.4%) | CK/CH/LSC/99I  (CK/CH/LSC/99I-type)  (96.5%) | RDP, GENECONV, Bootscan, MaxChi, Chimera, 3Seq |
|  | 14175 | 15479 | Nsp11/12, Nsp13 | KX236007_ck/CH/LSD/100408_2010_Shandong (LX4-type)  (97.7%) | CK/CH/LSC/99I  (CK/CH/LSC/99I-type)  (98.9%) | RDP, GENECONV, Bootscan, MaxChi, Chimera, 3Seq |
| JF893452_YN_2005_Yunnan | 674 | 4836 | Nsp2, Nsp3 | Partridge_GD_S14_2003 (LX4-type)  (unknown) | KF411041_CK/CH/LGX/091109_2009_Guangxi (LX4-type)  (94.5%) | RDP, GENECONV, Bootscan, MaxChi, Chimera, 3Seq |
|  | 12802 | 14301 | Nsp11/12 | CK/CH/LSC/99I  (CK/CH/LSC/99I-type)  (96%) | KF853202_SDZB0808_2008_Shandong (LX4-type)  (99.1%) | RDP, GENECONV, Bootscan, MaxChi, Chimera, 3Seq |
| EU637854_CK/CH/LSD/05I_2005_Shandong | 1 | 3758 | 5'UTR, Nsp2,  Nsp3 | MG448607_CK/CH/LHB/121042_2012_Hebei (Taiwan-I-type)  (92.7%) | Conn46_1972  (Conn-type)  (96.2%) | RDP, GENECONV, Bootscan, MaxChi, Chimera, 3Seq |
|  | 24600 | 27220 | Membrane, 4b, 4c, 5a, 5b, Nucleocapsid, 6b | Georgia  (unknown) | Conn46_1972  (Conn-type)  (99.6%) | RDP, GENECONV, Bootscan, MaxChi, Chimera, 3Seq |
| KX252779_ck/CH/LLN/06I_2006_Liaoning | 1 | 2752 | 5'UTR, Nsp2,  Nsp3 | JX840411_YX10_2010_Zhejiang (LX4-type)  (94.3%) | Partridge_GD_S14_2003 (LX4-type)  (96.8%) | RDP, GENECONV, Bootscan, MaxChi, Chimera, 3Seq |
|  | 9170 | 10132 | Nsp5, Nsp6 | KX252772_ck/CH/LSHH/03II_2003_Shanghai (LX4-type)  (95.2%) | Lowa  (95.4%) | RDP, GENECONV, MaxChi, Chimera, 3Seq |
|  | 23699 | 24760 | Spike, 3a, 3b, Envelope, Membrane | KX302868_ck/CH/LHLJ/130744_2013_Heilongjiang (LX4-type)  (88.5%) | KF663559_ck/CH/IBTZ/2012_Jiangsu (LX4-type)  (99%) | RDP, GENECONV, Bootscan, MaxChi, Chimera |
| HQ850618_GX-YL9_2007_Guangxi | 4454 | 8175 | Nsp3, Nsp4 | KX252788_ck/CH/LHLJ/08-6_2008_Heilongjiang (LX4-type)  (unknown) | KX219793_ck/CH/LHB/111168_2011_Hebei (LX4-type)  (98.6%) | RDP, GENECONV, Bootscan, MaxChi, Chimera, 3Seq |
|  | 22100 | 27560 | Spike, 3a, 3b, Envelope, Membrane, 4b, 4c, 5a, 5b, Nucleocapsid, 6b, 3'UTR | KU317090_SAIBK2_2011_Sichuan (LX4-type)  (96.7%) | JF893452_YN_2005_Yunnan (CK/CH/LSC/99I-type)  (98.3%) | RDP, GENECONV, Bootscan, MaxChi, Chimera, 3Seq |
| KX219791_ck/CH/LHLJ/07I_2007_Heilongjiang | 1 | 25019 | 5'UTR, Nsp2, Nsp3, Nsp4, Nsp5, Nsp6, Nsp7, Nsp8, Nsp9, Nsp10, Nsp11/12, Nsp13, Nsp14, Nsp15, Nsp16, Spike, 3a, 3b, Envelope, Membrane | CK/CH/LSC/99I  (CK/CH/LSC/99I-type)  (95.8%) | KX375805_ck/CH/LJS/07V_2007_Jiangsu (LX4-type)  (99.5%) | RDP, MaxChi, 3Seq |
| KX252777_ck/CH/LSD/07I_2007_Shandong | 1979 | 2734 | Nsp2, Nsp3 | KX252788_ck/CH/LHLJ/08-6_2008_Heilongjiang (LX4-type)  (99%) | CK/CH/LSC/99I  (CK/CH/LSC/99I-type)  (96.3%) | RDP, GENECONV, MaxChi, Chimera, 3Seq |
|  | 18107 | 19837 | Nsp14, Nsp15,  Nsp16 | KX275393_ck/CH/LHLJ/130705_2013_Heilongjiang (LX4-type)  (98.4%) | KX302875_ck/CH/LJL/100512_2010_Jiangsu (LX4-type)  (99.3%) | RDP, GENECONV, MaxChi, Chimera, 3Seq |
| KX364296_ck/CH/LJL/08-9_2008_Jilin | 22110 | 24848 | Spike, 3a, 3b,  Envelope, Membrane | KX302869_ck/CH/LHLJ/090806_2009_Heilongjiang (LX4-type)  (98.3%) | Peafowl_GD_KQ6_2003 (Peafowl/GD/KQ6/2003-type)  (99.7%) | RDP, GENECONV, Bootscan, MaxChi, Chimera, 3Seq |
| KX275394_ck/CH/LSD08-8_2008_Shandong | 1706 | 2924 | Nsp2, Nsp3 | KX219792_ck/CH/LGS/06I_2006_Gansu (LX4-type)  (90.1%) | CK/CH/LSC/99I  (CK/CH/LSC/99I-type)  (97.8%) | RDP, GENECONV, Bootscan, MaxChi, 3Seq |
|  | 26848 | 27654 | Nucleocapsid,  6b, 3'UTR, | KX252777_ck/CH/LSD/07I_2007_Shandong (LX4-type)  (98.6%) | Georgia  (96.2%) | RDP, GENECONV, Bootscan, MaxChi, Chimera, 3Seq |
| KP118894_ck/CH/LGD/090907_2009_Guangdong | 4206 | 8206 | Nsp3, Nsp4 | KX252788_ck/CH/LHLJ/08-6_2008_Heilongjiang (LX4-type)  (unknown) | KX219793_ck/CH/LHB/111168_2011_Hebei (LX4-type)  (99.3%) | RDP, GENECONV, Bootscan, MaxChi, Chimera, 3Seq |
|  | 20986 | 22200 | Spike | KU317090_SAIBK2_2011_Sichuan (LX4-type)  (95.3%) | KP118893_ck/CH/LSD/110410_2011_Shandong (4/91-type)  (97%) | RDP, GENECONV, MaxChi, Chimera, 3Seq |
| KF411041_CK/CH/LGX/091109_2009_Guangxi | 20438 | 21838 | Spike | KX302874_ck/CH/LGS/131148_2013_Gansu (LX4-type)  (96.7%) | SAIBK  (CK/CH/LSC/99I-type)  (95.9%) | RDP, GENECONV, MaxChi, Chimera, 3Seq |
| KX252783_ck/CH/LHLJ/090510_2009_Heilongjiang | 1968 | 2746 | Nsp2, Nsp3 | KX302869_ck/CH/LHLJ/090806_2009_Heilongjiang (LX4-type)  (99.1%) | CK/CH/LSC/99I  (CK/CH/LSC/99I-type)  (95.9%) | RDP, GENECONV, Bootscan, MaxChi, Chimera, 3Seq |
| KX252784_ck/CH/LLN/090910_2009_Liaoning | 18104 | 19834 | Nsp14, Nsp15,  Nsp16 | KX275393_ck/CH/LHLJ/130705_2013_Heilongjiang (LX4-type)  (97.9%) | KX302875_ck/CH/LJL/100512_2010_Jiangsu (LX4-type)  (99.8%) | RDP, GENECONV, Bootscan, MaxChi, Chimera, 3Seq |
| KX219794_ck/CH/LJS/101109_2010_Jiangsu | 18773 | 19782 | Nsp15, Nsp16 | KX259252_ck/CH/LJS/110439_2011_Jiangsu (LX4-type)  (92.5%) | KP118893_ck/CH/LSD/110410_2011_Shandong (LX4-type)  (100%) | RDP, GENECONV, Bootscan, MaxChi, Chimera, 3Seq |
| KX236007_ck/CH/LSD/100408_2010_Shandong | 6018 | 6677 | Nsp3 | KX236002_ck/CH/LSD/111037_2011_Shandong (LX4-type)  (98.7%) | BJ (LX4-type)  (98%) | RDP, GENECONV, Bootscan, MaxChi, Chimera, 3Seq |
|  | 23035 | 25096 | Spike, 3a, 3b,  Envelope, Membrane | KX236002_ck/CH/LSD/111037_2011_Shandong (LX4-type)  (98.3%) | Partridge_GD/S14/2003  (LX4-type)  (99.8%) | RDP, GENECONV, MaxChi, Chimera, 3Seq |
| KX252785_ck/CH/LSD/100311_2010_Shandong | 8183 | 9252 | Nsp4, Nsp5 | KX252791_ck/CH/LLN/98I_1998_Liaoning (LX4-type)  (94.3%) | KP118887_ck/CH/LHB/140532_2014_Hebei (4/91-type)  (99.1%) | RDP, GENECONV, Bootscan, MaxChi, Chimera, 3Seq |
| JX840411_YX10_2010_Zhejiang | 26344 | 26864 | Nucleocapsid | KX364293_ck/CH/LJL/07III_2007_Jilin (LX4-type)  (98%) | CK_CH_LSC_99I  (CK/CH/LSC/99I-type)  (97.3%) | RDP, GENECONV, MaxChi, Chimera, 3Seq |
| KJ425489_ck/CH/LHB/111172_2011_Hebei | 24152 | 25352 | Envelope, Membrane, 4b | KJ425497_ck/CH/LHB/130598_2013_Hebei (Mass-type)  (99.8%) | JF330898_ck/CH/LHB/100801_2010_Hebei (Taiwan-I-type )  (99.9%) | RDP, GENECONV, Bootscan, MaxChi, Chimera, 3Seq |
| KX434790_ck/CH/LJS/111210_2011_Jiangsu | 1 | 6727 | 5'UTR, Nsp2,  Nsp3 | KX348114_ck/CH/LDL/05III_2005_Liaoning (LX4-type)  (92.3%) | KP036502_ck/CH/LHLJ/140906_2014_Heilongjiang (4/91-type)  (99.3%) | RDP, GENECONV, Bootscan, MaxChi, Chimera, 3Seq |
|  | 9602 | 13591 | Nsp5, Nsp6, Nsp7, Nsp8, Nsp9, Nsp10, Nsp11/12 | KF853202_SDZB0808_2008_Shandong (LX4-type)  (99.1%) | KF663559_ck/CH/IBTZ/2012_Jiangsu (LX4-type)  (99.2%) | RDP, GENECONV, MaxChi, Chimera, 3Seq |
| KC506155_ck/CH/LJL/111054_2011_Jilin | 2763 | 3653 | Nsp3 | KJ425502_ck/CH/LHB/131143_2013_Hebei (Mass-type)  (99.9%) | Conn46_1972  (Conn-type)  (100%) | RDP, GENECONV, Bootscan, MaxChi, Chimera, 3Seq |
|  | 25155 | 27581 | 4b, 4c, 5a, 5b, Nucleocapsid, 6b, 3'UTR | JF828981_ck/CH/LDL/101212_2010_Liaoning (Mass-type)  (unknown) | Conn46_1972  (Conn-type)  (99.9%) | RDP, GENECONV, Bootscan, MaxChi, Chimera, 3Seq |
| KF411040_CK/CH/LLN/111169_2011_Liaoning | 564 | 3455 | Nsp2, Nsp3 | KX219796_ck/CH/LXJ/111265_2011_Xinjiang (LX4-type)  (96%) | KX219793_ck/CH/LHB/111168_2011_Hebei (LX4-type)  (99.1%) | RDP, GENECONV, Bootscan, MaxChi, Chimera, 3Seq |
|  | 5415 | 7278 | Nsp3 | KX434788_ck/CH/LHLJ/130622_2013_Heilongjiang (LX4-type)  (99.4%) | KX259252_ck/CH/LJS/110439_2011_Jiangsu (LX4-type)  (99.6%) | RDP, GENECONV, Bootscan, 3Seq |
|  | 18282 | 26127 | Nsp14, Nsp15, Nsp16, Spike, 3a, 3b, Envelope, Membrane, 4b, 4c, 5a, 5b, Nucleocapsid | KX236013_ck/CH/LSD/120742_2012_Shandong (LX4-type)  (97%) | KJ425485_ck/CH/LDL/110931_2011_Liaoning (Mass-type)  (97.9%) | RDP, GENECONV, Bootscan, MaxChi, Chimera, 3Seq |
| KJ425485_ck/CH/LDL/110931_2011_Liaoning | 24154 | 27528 | Envelope, Membrane, 4b, 4c, 5a, 5b, Nucleocapsid, 6b, 3'UTR | JF828981_ck/CH/LDL/101212_2010_Liaoning (Mass-type)  (99.3%) | Conn46_1972  (Conn-type)  (99.9%) | RDP, GENECONV, Bootscan, MaxChi, Chimera, 3Seq |
| KP118885_ck/CH/LSD/110857_2011_Shandong | 1 | 4811 | 5'UTR, Nsp2,  Nsp3 | Gray  (unknown) | KP868573_CK/CH/LJL/130908_2013_Jilin (Conn-type)  (99.7%) | RDP, GENECONV, Bootscan, MaxChi, Chimera, 3Seq |
|  | 9554 | 15598 | Nsp5, Nsp6, Nsp7, Nsp8, Nsp9, Nsp10, Nsp11/12, Nsp13 | KP118881_ck/CH/LBJ/140413_2014_Beijing (4/91-type)  (99.9%) | KJ425485_ck/CH/LDL/110931_2011_Liaoning (Mass-type)  (100%) | RDP, GENECONV, Bootscan, MaxChi, Chimera, 3Seq |
| KJ435283_ck/CH/LSD/111219_2011_Shandong | 18128 | 24076 | Nsp14, Nsp15, Nsp16, Spike, 3a, 3b | KF663559_ck/CH/IBTZ/2012_Jiangsu (LX4-type)  (95.4%) | KJ435286_ck/CH/LSD/1112150_2011_Shandong (Peafowl/GD/KQ6/2003-type)  (96.1%) | RDP, GENECONV, Bootscan, MaxChi, Chimera, 3Seq |
| KU317090_SAIBK2_2011_Sichuan | 5804 | 8098 | Nsp3, Nsp4 | KX219792_ck/CH/LGS/06I_2006_Gansu (LX4-type)  (93.7%) | JF893452_YN_2005_Yunnan (CK/CH/LSC/99I-type)  (98.1%) | RDP, Bootscan, MaxChi, Chimera, 3Seq |
| JX195176_ck/CH/LZJ/111113_2011_Zhejiang | 19390 | 21826 | Nsp15, Nsp16, Spike | KX348116_ck/CH/LBJ/140514_2014_Beijing (LX4-type)  (97.3%) | KP118891_ck/CH/LHLJ/111246_2011_Heilongjiang (4/91-type)  (99.3%) | RDP, GENECONV, Bootscan, MaxChi, Chimera, 3Seq |
| KX348115_ck/CH/LAH/120907_2012_Anhui | 21561 | 22451 | Spike | KX259254_ck/CH/LSD/090314_2009_Shandong (LX4-type)  (97.1%) | TW2575/98  (Taiwan-I-type)  (98.8%) | RDP, GENECONV, Bootscan, MaxChi, Chimera, 3Seq |
| KC119407_Ck/CH/LGD/120724_2012_Guangdong | 1 | 5271 | 5'UTR, Nsp2,  Nsp3 | KX219796_ck/CH/LXJ/111265_2011_Xinjiang (LX4-type)  (95.7%) | KX219793_ck/CH/LHB/111168_2011_Hebei (LX4-type)  (99.1%) | RDP, GENECONV, Bootscan, MaxChi, Chimera, 3Seq |
|  | 21044 | 22139 | Spike | KU317090_SAIBK2_2011_Sichuan (LX4-type)  (95.7%) | KP118893_ck/CH/LSD/110410_2011_Shandong (4/91-type)  (98.4%) | RDP, GENECONV, MaxChi, Chimera, 3Seq |
| KF663559_ck/CH/IBTZ/2012_Jiangsu | 26209 | 26854 | Nucleocapsid | KF668605_CK/CH/SD09/005_2009_Shandong (LX4-type)  (97.6%) | KX252774_ck/CH/LHB/130589_2013_Hebei (LX4-type)  (98.9%) | RDP, GENECONV, MaxChi, Chimera, 3Seq |
| KJ128295_CK/CH/SD/121220_2012_Shandong | 195 | 25359 | 5'UTR, Nsp2, Nsp3, Nsp4, Nsp5, Nsp6, Nsp7, Nsp8, Nsp9, Nsp10, Nsp11/12, Nsp13, Nsp14, Nsp15, Nsp16, Spike, 3a, 3b, Envelope, Membrane, 4b | KX364294_ck/CH/LBJ/140528_2014_Beijing (LX4-type)  (unknown) | KX219799_ck/CH/LSD/130211_2013_Shandong (LX4-type)  (99.8%) | RDP, GENECONV, MaxChi, 3Seq |
| KX219798_ck/CH/LSD/120913_2012_Shandong | 19664 | 20236 | Nsp16 | KX247127_ck/CH/LHB/111190_2011_Hebei (LX4-type)  (99.2%) | KP118893_ck/CH/LSD/110410_2011_Shandong (4/91-type)  (100%) | RDP, GENECONV, MaxChi, Chimera, 3Seq |
| KX219801_ck/CH/LSD/120437_2012_Shandong | 9169 | 10356 | Nsp5, Nsp6 | KX259253_ck/CH/LHN/120338_2012_Hunan (LX4-type)  (unknown) | KX372249_ck/CH/LHLJ/121219_2012_Heilongjiang (LX4-type)  (99.6%) | RDP, GENECONV, Bootscan, MaxChi, Chimera |
| KP343691_ck/CH/LGX/130530_2013_Guangxi | 1 | 17081 | 5'UTR, Nsp2, Nsp3, Nsp4, Nsp5, Nsp6, Nsp7, Nsp8, Nsp9, Nsp10, Nsp11/12, Nsp13, Nsp14 | IBV-EP3  (unknown) | KJ425501_ck/CH/LHB/131142_2013_Hebei (Mass-type)  (99.9%) | RDP, GENECONV, Bootscan, MaxChi, Chimera, 3Seq |
|  | 20919 | 22036 | Spike | KX252785_ck/CH/LSD/100311_2010_Shandong (LX4-type)  (unknown) | CK/CH/LSC/99I (CK/CH/LSC/99I-type)  (97.5%) | RDP, GENECONV, Bootscan, MaxChi, Chimera, 3Seq |
| KJ425496_ck/CH/LHB/130573_2013_Hebei | 1 | 18988 | 5'UTR, Nsp2, Nsp3, Nsp4, Nsp5, Nsp6, Nsp7, Nsp8, Nsp9, Nsp10, Nsp11/12, Nsp13, Nsp14, Nsp15 | Partridge/GD/S14/2003  (LX4-type)  (99.8%) | KJ425501_ck/CH/LHB/131142_2013_Hebei (Mass-type)  (99.9%) | RDP, GENECONV, Bootscan, MaxChi, Chimera, 3Seq |
| KJ425497_ck/CH/LHB/130598_2013_Hebei | 25353 | 27514 | 4b, 4c, 5a, 5b, Nucleocapsid, 6b, 3'UTR | KJ425499_ck/CH/LHB/131118_2013_Hebei (Mass-type)  (99.9%) | KP118881_ck/CH/LBJ/140413_2014_Beijing (4/91-type)  (99.9%) | RDP, GENECONV, Bootscan, MaxChi, Chimera, 3Seq |
| KP118889_ck/CH/LHB/130575_2013_Hebei | 19211 | 21826 | Nsp15, Nsp16, Spike | KX348116_ck/CH/LBJ/140514_2014_Beijing (LX4-type)  (97.8%) | KP118891_ck/CH/LHLJ/111246_2011_Heilongjiang (4/91-type)  (99.3%) | RDP, GENECONV, Bootscan, MaxChi, Chimera, 3Seq |
| KX252776_ck/CH/LHB/131144_2013_Hebei | 22137 | 26269 | Spike, 3a, 3b, Envelope, Membrane, 4b, 4c, 5a, 5b, Nucleocapsid | KP118894_ck/CH/LGD/090907_2009_Guangdong (LX4-type)  (97.2%) | KX252775_ck/CH/LHB/130628_2013_Hebei (LX4-type)  (99.7%) | RDP, GENECONV, Bootscan, MaxChi, Chimera, 3Seq |
| KX275393_ck/CH/LHLJ/130705_2013_Heilongjiang | 8282 | 10876 | Nsp4, Nsp5, Nsp6, Nsp7 | KX400753_ck/CH/LHB/110123_2011_Hebei (LX4-type)  (98.9%) | KX252787_ck/CH/LLN/131040_2013_Liaoning (LX4-type)  (99.7%) | RDP, GENECONV, Bootscan, MaxChi, Chimera, 3Seq |
| KX302868_ck/CH/LHLJ/130744_2013_Heilongjiang | 26152 | 27542 | Nucleocapsid, 6b, 3'UTR | KX372249_ck/CH/LHLJ/121219_2012_Heilongjiang (LX4-type)  (99.8%) | KP036502_ck/CH/LHLJ/140906_2014_Heilongjiang (4/91-type)  (98.9%) | RDP, GENECONV, Bootscan, Chimera, 3Seq |
| KX302865_ck/CH/LJS/131102_2013_Jiangsu | 10202 | 12615 | Nsp6, Nsp7, Nsp8, Nsp9, Nsp10, Nsp11/12 | KX259257_ck/CH/LJS/120848_2012_Jiangsu (LX4-type)  (97.3%) | KX375806_ck/CH/LHLJ/110673_2011_Heilongjiang (LX4-type)  (99.7%) | RDP, GENECONV, Bootscan, MaxChi, Chimera, 3Seq |
| KP036505_ck/CH/LJL/130925_2013_Jilin | 20446 | 21738 | Spike | KJ128295_CK/CH/SD/121220_2012_Shandong (LX4-type)  (96.6%) | KP118880_ck/CH/LHB/130927_2013_Hebei (4/91-type)  (98%) | RDP, GENECONV, Bootscan, MaxChi, Chimera, 3Seq |
| KX364297_ck/CH/LJL/131006_2013_Jilin | 18503 | 20556 | Nsp15, Nsp16, Spike | KX219798_ck/CH/LSD/120913_2012_Shandong (LX4-type)  (96.6%) | KU356856_ck/CH/SCYB/140913_2014_Sichuan (4/91-type)  (100%) | RDP, GENECONV, Bootscan, MaxChi, Chimera, 3Seq |
| KX219800_ck/CH/LSD/130611_2013_Shandong | 3923 | 7314 | Nsp3 | KX252788_ck/CH/LHLJ/08-6_2008_Heilongjiang (LX4-type)  (unknown) | KX219793_ck/CH/LHB/111168_2011_Hebei (LX4-type)  (98.2%) | RDP, GENECONV, Bootscan, MaxChi, Chimera, 3Seq |
|  | 17486 | 23230 | Nsp14, Nsp15, Nsp16, Spike | KX252773_ck/CH/LHB/140542_2014_Hebei (LX4-type)  (97.8%) | KX247127_ck/CH/LHB/111190_2011_Hebei (LX4-type)  (99.6%) | RDP, Bootscan, MaxChi, Chimera, 3Seq |
| KX348116_ck/CH/LBJ/140514_2014_Beijing | 6414 | 9122 | Nsp3, Nsp4,  Nsp5 | KX364295_ck/CH/LBJ/140511_2014_Beijing (LX4-type)  (99.8%) | KX372249_ck/CH/LHLJ/121219_2012_Heilongjiang (LX4-type)  (99.6%) | RDP, GENECONV, MaxChi, Chimera, 3Seq |
|  | 23568 | 26064 | Spike, 3a, 3b, Envelope, Membrane, 4b, 4c, 5a, 5b, Nucleocapsid | KX275393_ck/CH/LHLJ/130705_2013_Heilongjiang (LX4-type)  (unknown) | KX247127_ck/CH/LHB/111190_2011_Hebei (LX4-type)  (99.6%) | RDP, GENECONV, Bootscan, MaxChi, Chimera, 3Seq |
| KX364294_ck/CH/LBJ/140528_2014_Beijing | 25367 | 27337 | 4b, 4c, 5a, 5b, Nucleocapsid, 6b | JX840411_YX10_2010_Zhejiang  (LX4-type)  (97.3%) | KX236008_ck/CH/LDL/130325_2013_Liaoning (LX4-type)  (99.6%) | RDP, GENECONV, Bootscan, Chimera, 3Seq |
| KP118887_ck/CH/LHB/140532_2014_Hebei | 5 | 4768 | 5'UTR, Nsp2,  Nsp3 | Gray  (unknown) | KP868573_CK/CH/LJL/130908_2013_Jilin (Conn-type)  (99.9%) | RDP, GENECONV, Bootscan, MaxChi, Chimera, 3Seq |
| KP790146_CK/CH/LHLJ/140901_2014_Heilongjiang | 5520 | 9994 | Nsp3, Nsp4, Nsp5, Nsp6 | KP790145_CK/CH/LHLJ/141105_2014_Heilongjiang (LX4-type)  (98.6%) | KP790144_CK/CH/LHLJ/140756_2014_Heilongjiang (LX4-type)  (99.4%) | RDP, GENECONV, Bootscan, MaxChi, Chimera, 3Seq |
|  | 19626 | 21828 | Nsp16, Spike | KX275390_ck/CH/LHLJ/141103_2014_Heilongjiang (LX4-type)  (98.7%) | TW257598  (Taiwan-I-type)  (98.2%) | RDP, GENECONV, Bootscan, MaxChi, Chimera, 3Seq |
| KX425847_ck/CH/LJL/140734_2014_Jilin | 26702 | 27675 | Nucleocapsid, 6b, 3'UTR | KX302867_ck/CH/LJL/150430_2015_Jilin (LX4-type)  (98.8%) | KJ425500_ck/CH/LHB/131132_2013_Hebei (Mass-type)  (97.3%) | RDP, GENECONV, Bootscan, MaxChi, Chimera, 3Seq |
| KP790143_CK/CH/LDL/140520_2014_Liaoning | 20347 | 22451 | Nsp16, Spike | KX302874_ck/CH/LGS/131148_2013_Gansu (LX4-type)  (98.3%) | TW257598  (Taiwan-I-type)  (98.7%) | RDP, GENECONV, Bootscan, MaxChi, Chimera, 3Seq |
| KU356856_ck/CH/SCYB/140913_2014_Sichuan | 1 | 15866 | 5'UTR, Nsp2, Nsp3, Nsp4, Nsp5, Nsp6, Nsp7, Nsp8, Nsp9, Nsp10, Nsp11/12, Nsp13 | Beaudette_CK  (unknown) | KJ425493vck/CH/LHB/120749_2012_Hebei (Mass-type)  (98.5%) | RDP, GENECONV, Bootscan, MaxChi, Chimera |
|  | 20942 | 22079 | Spike | KX252785_ck/CH/LSD/100311_2010_Shandong (LX4-type)  (93.2%) | CK/CH/LSC/99I (CK/CH/LSC/99I-type)  (97.8%) | RDP, GENECONV, Bootscan, MaxChi, Chimera, 3Seq |
| KX302867_ck/CH/LJL/150430_2015_Jilin | 19374 | 21065 | Nsp15, Nsp16, Spike | JX195176_ck/CH/LZJ/111113_2011_Zhejiang (LX4-type)  (98.5%) | KX236014_ck/CH/LHB/120497_2014_Hebei (LX4-type)  (98.9%) | RDP, GENECONV, Bootscan, MaxChi |
| MG197727_CK/CH/GD/QY16_2016_Guangdong | 20337 | 21826 | Nsp16, Spike | KX348115_ck/CH/LAH/120907_2012_Anhui (LX4-type)  (97.6%) | KP118890_ck/CH/LHB/130578_2013_Hebei (LX4-type)  (99%) | RDP, GENECONV, Bootscan, MaxChi |

^a^ Only transferred gene fragments where *p* ≤ 1 × 10^−12^ are included in the table. ^b^ Genes indicate the coding sequences contained within the fragment introduced by recombination. ^c^ Major parent = Sequence most closely related to the sequence surrounding the transferred fragment. ^d^ Minor parent = Sequence closely related to the transferred fragment in the recombinant. ^e^ Unknown = only one parent and a recombinant need be in the alignment for a transferred fragment to be detectable.
