# Supplementary material for: Phylogenetic and Spatiotemporal Analyses of the Complete Genome Sequences of Avian Coronavirus Infectious Bronchitis Virus in China During 1985–2020: Revealing Coexistence of Multiple Transmission Chains and the Origin of LX4-Type Virus
Source: Front Microbiol. 2022 Apr 4;13:693196. doi: 10.3389/fmicb.2022.693196 (PMC9013971; doi:10.3389/fmicb.2022.693196)
Supplement: Supplementary Figure 1 — Distribution of 212 IBV isolates in China. Distribution is presented as the number of isolates per province or autonomous city (zone), from most cases (45, dark red) to no case (0, white). [file Data_Sheet_1.ZIP › Supplementary material/Supplementary Table 8.doc]

**Supplementary Table 8. Genetic recombination events of the S1 gene of IBV isolates detected by RDP 4 software.**

| **Potential**  **recombinant** | | **Breakpoints** | | | | **Major parent (similarity)** | | **Minor parent (similarity)** | | **Detection method ^a^** |
| --- | --- | --- | --- | --- | --- | --- | --- | --- | --- | --- |
|  |  | **Beginning** | | **Ending** | |  |  |  |  |  |
| EU637854_CK/CH/LSD/05I_2005_Shandong | 82 | | 643 | | KX077987_ck/CH/LDL/150434-III_2015_Liaoning (Taiwan-Ⅰ-type)  (91.5%) | | KF853202_SDZB0808_2008_Shandong (LX4-type)  (82.7%) | | MaxChi, Chimera, 3Seq | |
| KF411041_CK/CH/LGX/091109_2009_Guangxi | 1502 | | 1626 | | JF893452_YN_2005_Yunnan (CK/CH/LSD/99I-type)  (98.2%) | | MG197727_CK/CH/GD/QY16_2016_Guangdong (4/91-type)  (99.2%) | | RDP, GENECONV, Bootscan, MaxChi, Chimera, 3Seq | |
| KX259251_ck/CH/LJS/101237_2010_Jiangsu | 859 | | 1494 | | KX236003_ck/CH/LSD/111025_2011_Shandong (LX4-type)  (99%) | | KX219792_ck/CH/LGS/06I_2006_Gansu (LX4-type)  (99.2%) | | Bootscan, Chimera, 3Seq | |
| KX640829_ck/CH/LGX/111119_2011_Guangxi | 599 | | 1101 | | KX302873_ck/CH/LJL/140924_2014_Jilin (LX4-type)  (92.5%) | | HQ850618_GX-YL9_2007_Guangxi (LX4-type)  (91.8%) | | RDP, Bootscan, MaxChi, Chimera, 3Seq | |
| KP118886_ck/CH/LSD/111235_2011_Shandong | 688 | | 1417 | | KF411041_CK/CH/LGX/091109_2009_Guangxi (CK/CH/LSD/99I-type)  (95.8%) | | KP036505_ck/CH/LJL/130925_2013_Jilin (4/91-type)  (97.1%) | | RDP, GENECONV, Bootscan, MaxChi, Chimera, 3Seq | |
| KX348115_ck/CH/LAH/120907_2012_Anhui | 1008 | | 1444 | | TW2575/98 (Taiwan-Ⅰ-type)  (76%) | | KX259251_ck/CH/LJS/101237_2010_Jiangsu (LX4-type)  (96.3%) | | RDP, GENECONV, Bootscan, MaxChi | |
| KC013541_CK/CH/LGD/120723_2012_Guangdong | 678 | | 1624 | | JF893452_YN_2005_Yunnan (CK/CH/LSD/99I-type)  (97.9%) | | KP118892_ck/CH/LLN/130101_2013_Liaoning (4/91-type)  (98.7%) | | RDP, GENECONV, Bootscan, MaxChi, Chimera, 3Seq | |
| KJ425496_ck/CH/LHB/130573_2013_Hebei | 1365 | | 1611 | | KJ425485_ck/CH/LDL/110931_2011_Liaoning (Mass-type)  (99.6%) | | SAIBK (CK/CH/LSD/99I-type)  (99.6%) | | RDP, GENECONV, Bootscan, 3Seq | |
| KP118890_ck/CH/LHB/130578_2013_Hebei | 1455 | | 1617 | | KP118892_ck/CH/LLN/130101_2013_Liaoning (4/91-type)  (99.5%) | | KX389094_ck/CH/LBJ/120481_2012_Beijing (LX4-type)  (100%) | | RDP, GENECONV, Bootscan, MaxChi, Chimera, 3Seq | |
| KX434788_ck/CH/LHLJ/130622_2013_Heilongjiang | 1202 | | 1307 | | JX195175_ck/CH/LDL/091022_2009_Liaoning (LX4-type)  (98.4%) | | MG197727_CK/CH/GD/QY16_2016_Guangdong (4/91-type)  (100%) | | RDP, GENECONV, Bootscan, 3Seq | |
| KP036505_ck/CH/LJL/130925_2013_Jilin | 74 | | 1368 | | KX219795_ck/CH/LSD/150311_2015_Shandong (LX4-type)  (95.8%) | | MG197727_CK/CH/GD/QY16_2016_Guangdong (4/91-type)  (97.8%) | | RDP, 3Seq | |
| KP036502_ck/CH/LHLJ/140906_2014_Heilongjiang | 595 | | 1555 | | JF274479_ck/CH/LHLJ/07VII_2007_Heilongjiang (Mass-type)  (97.1%) | | MG197727_CK/CH/GD/QY16_2016_Guangdong (LX4-type)  (97.5%) | | RDP, GENECONV, Bootscan, MaxChi, 3Seq | |
| KP790145_CK/CH/LHLJ/141105_2014_Heilongjiang | 1470 | | 1620 | | KP790144_CK/CH/LHLJ/140756_2014_Heilongjiang (Taiwan-Ⅰ-type)  (97.5%) | | MG197727_CK/CH/GD/QY16_2016_Guangdong (LX4-type)  (98.7%) | | RDP, GENECONV, Bootscan, MaxChi, Chimera, 3Seq | |
| KX434789_ck/CH/LJL/140530_2014_Jilin | 687 | | 1620 | | KT736032_ckCHLDL150434-II_2015_Liaoning (LDT3-A-type)  (97.5%) | | KP036505_ck/CH/LJL/130925_2013_Jilin (4/91-type)  (99.4%) | | RDP, GENECONV, Bootscan, MaxChi, Chimera, 3Seq | |
| KX236015_ck/CH/LDL/140709_2014_Liaoning | 629 | | 705 | | KX372249_ck/CH/LHLJ/121219_2012_Heilongjiang (LX4-type)  (97%) | | JF893452_YN_2005_Yunnan (CK/CH/LSD/99I -type)  (98.7%) | | RDP, Bootscan | |
| KX302867_ck/CH/LJL/150430_2015_Jilin | 1 | | 925 | | KP118890_ck/CH/LHB/130578_2013_Hebei (4/91-type)  (99.1%) | | KX348115_ck/CH/LAH/120907_2012_Anhui (LX4-type)  (97%) | | RDP, GENECONV, Bootscan, MaxChi, 3Seq | |

^a^ Only transferred gene fragments where *p* ≤ 1 × 10^−6^ are included in the table. The major parent is the sequence most closely related to that surrounding the transferred fragment. The minor parent is most closely related to the transferred fragment in the recombinant.
