# Supplementary material for: Phylogenetic and Spatiotemporal Analyses of the Complete Genome Sequences of Avian Coronavirus Infectious Bronchitis Virus in China During 1985–2020: Revealing Coexistence of Multiple Transmission Chains and the Origin of LX4-Type Virus
Source: Front Microbiol. 2022 Apr 4;13:693196. doi: 10.3389/fmicb.2022.693196 (PMC9013971; doi:10.3389/fmicb.2022.693196)
Supplement: Supplementary Figure 1 — Distribution of 212 IBV isolates in China. Distribution is presented as the number of isolates per province or autonomous city (zone), from most cases (45, dark red) to no case (0, white). [file Data_Sheet_1.ZIP › Supplementary material/Supplementary Table 9.doc]

**Supplementary Table 9.** The estimation of dN/dS ratios of S1, S2, E, M and N proteins of IBV isolates.

| Model | S1 protein | | | S2 protein | | | E protein | | | M protein | | | N protein | | |
| --- | --- | --- | --- | --- | --- | --- | --- | --- | --- | --- | --- | --- | --- | --- | --- |
|  | SLAC | FEL | IFEL | SLAC | FEL | IFEL | SLAC | FEL | IFEL | SLAC | FEL | IFEL | SLAC | FEL | IFEL |
| Mean dN/dS | 0.301 | | | 0.136 | | | 0.225 | | | 0.168 | | | 0.159 | | |
| Numbers of positive selection site | 30 | 22 | 29 | 8 | 10 | 10 | 1 | 4 | 6 | 5 | 5 | 4 | 12 | 13 | 13 |
| Numbers of neutral selection site | 299 | 244 | 258 | 335 | 214 | 258 | 70 | 54 | 57 | 133 | 112 | 127 | 191 | 160 | 177 |
| Numbers of purifying selection site | 226 | 289 | 268 | 283 | 402 | 358 | 40 | 53 | 48 | 88 | 109 | 95 | 206 | 236 | 219 |
| Positively selected sites (aa) | 19, 65, 100, 105, 117, 128, 153, 166, 171, 177, 288, 301, 404, 408, 451, 523 | | | 2, 31, 46, 82, 127, 407, 525, 546 | | | None | | | 47, 151 | | | 4, 6, 7, 46, 235, 322, 342, 346, 350, 356, 370 | | |

*SLAC (Single-likelihood ancestor counting), FEL (Fixed effects likelihood) and IFEL (Internal fixed effects likelihood).
